# Unraveling the Mystery of Insulin Resistance: From Principle Mechanistic Insights and Consequences to Therapeutic Interventions

**DOI:** 10.3390/ijms26062770

**Published:** 2025-03-19

**Authors:** Mohammad Muzaffar Mir, Mohammed Jeelani, Muffarah Hamid Alharthi, Syeda Fatima Rizvi, Shahzada Khalid Sohail, Javed Iqbal Wani, Zia Ul Sabah, Waad Fuad BinAfif, Partha Nandi, Abdullah M. Alshahrani, Jaber Alfaifi, Adnan Jehangir, Rashid Mir

**Affiliations:** 1 Department of Clinical Biochemistry, College of Medicine, University of Bisha, Bisha 61922, Saudi Arabia; 2 Department of Physiology, College of Medicine, University of Bisha, Bisha 61922, Saudi Arabia; mjeelani@ub.edu.sa; 3 Department of Family and Community Medicine, College of Medicine, University of Bisha, Bisha 61922, Saudi Arabia; mualharthi@ub.edu.sa (M.H.A.); partha@ub.edu.sa (P.N.);; 4 Department of Pathology, College of Medicine, University of Bisha, Bisha 61922, Saudi Arabia; sfatima@ub.edu.sa (S.F.R.); sksohail@ub.edu.sa (S.K.S.); 5 Department of Internal Medicine, College of Medicine, King Khalid University, Abha 61421, Saudi Arabia; drjiwani1959@gmail.com (J.I.W.); drziaulsabah@gmail.com (Z.U.S.); 6 Department of Internal Medicine, College of Medicine, University of Bisha, Bisha 61922, Saudi Arabia; waaeda@ub.edu.sa; 7 Department of Child Health, College of Medicine, University of Bisha, Bisha 61922, Saudi Arabia; jalfaifi@ub.edu.sa; 8 Biomedical Sciences Department, College of Medicine, King Faisal University, Al Ahsa 31982, Saudi Arabia; amalik@kfu.edu.sa; 9 Prince Fahd Bin Sultan Research Chair, Department of MLT, Faculty of Applied Medical Sciences, University of Tabuk, Tabuk 71491, Saudi Arabia; rashidmirtabuk@gmail.com

**Keywords:** insulin signaling, insulin resistance, signal transduction, T2DM, dyslipidemia, metabolic disease, lipotoxicity, inflammasome

## Abstract

Insulin resistance (IR) is a significant factor in the development and progression of metabolic-related diseases like dyslipidemia, T2DM, hypertension, nonalcoholic fatty liver disease, cardiovascular and cerebrovascular disorders, and cancer. The pathogenesis of IR depends on multiple factors, including age, genetic predisposition, obesity, oxidative stress, among others. Abnormalities in the insulin-signaling cascade lead to IR in the host, including insulin receptor abnormalities, internal environment disturbances, and metabolic alterations in the muscle, liver, and cellular organelles. The complex and multifaceted characteristics of insulin signaling and insulin resistance envisage their thorough and comprehensive understanding at the cellular and molecular level. Therapeutic strategies for IR include exercise, dietary interventions, and pharmacotherapy. However, there are still gaps to be addressed, and more precise biomarkers for associated chronic diseases and lifestyle interventions are needed. Understanding these pathways is essential for developing effective treatments for IR, reducing healthcare costs, and improving quality of patient life.

## 1. Introduction

As early as the 1930s, it was recognized that there existed patients who were severely hyperglycemic and/or diabetic yet were unresponsive to exogenous therapy with insulin. It was subsequently recognized that insulin-resistant states are characterized by resistance to the metabolic actions of insulin rather than true insulin insufficiency, and may coexist with either obesity and/or other features of the metabolic syndrome or acanthosis nigricans [1,2,3]. Insulin resistance (IR) predicts future weight gain, cardiovascular disease, and type 2 diabetes [4,5]. Whether obesity or IR is the primary defect, the two often coexist and clearly interact, and it is the combination of these two factors that appear particularly detrimental [1,6,7]. As the obesity epidemic worsens, it is also reasonable to anticipate even more cases of IR [8]. IR is a hallmark of the pathophysiology of prediabetes and metabolic syndrome [6,9]. In fact, IR has been defined as the cardinal manifestation of the metabolic syndrome [9,10]. At the molecular level, primarily in settings of obesity and obesity-related IR, there are clear physiologic underpinnings whereby metabolic intermediates exert inhibitory effects and can specifically block insulin signal transduction [6,7,8]

However, human genetic studies argue that IR can also be the initial hit that promotes obesity and associated sequelae [9,11]. If this dichotomy has any significance, it is to accurately realize that a deep and precise understanding of these notions can guide both the exact processes of how insulin signal transduction is altered and be successfully probed for therapeutic approaches [1,9].

## 2. Definition and Clinical Relevance

IR refers to a reduced ability of target tissues to respond to insulin, leading to impaired glucose uptake and, eventually, to the development of T2DM [6,8,9]. IR is widely recognized as a significant risk factor for many different disorders, including cardiovascular diseases, metabolic syndromes, nonalcoholic fatty liver disease, chronic kidney disease, inflammation, and age-related cognitive impairment [3,4,12,13,14,15]. Despite the high prevalence and large disease burden of IR, the mechanistic underpinnings of the syndrome and useful strategies for ameliorating or preventing its development are poorly understood and continue to be areas of intense research investigation [1,6].

The key hallmark of IR is impaired insulin-mediated glucose disposal by peripheral microvascular endothelium, adipose tissue, and skeletal muscle in response to the metabolic needs of the whole body [1,2,6]. The primary etiological factor that leads to the development of IR is the maladaptation of various intrinsically interconnected physiological processes that together govern whole-body glucose homeostasis [9,15]. Available evidence suggests that IR and the associated vascular and neuronal dysfunctions are likely to develop in parallel [8,16,17].

More importantly, IR and its related disorders often coexist, and patients with IR are prone to more rapid progression of these various conditions [1,10]. Here, we review important basic knowledge about the biochemical and molecular mechanisms that have a role in causing and maintaining IR. These mechanisms range from abnormalities in the transport of plasma glucose into peripheral microvascular endothelium, adipose tissue, and skeletal muscle to mitochondrial oxidative stress [1,6,9]. Certain forms of IR are known to develop de novo, while others manifest themselves as part of a complex syndrome. Although the correlation between T2DM and insulin resistance is well-documented, other forms of diabetes may not exhibit this association [1,7].

Of the well-established factors that contribute to IR, obesity related to lipid-overloaded conditions, with enhanced release of fatty acids from adipose tissue in plasma, associated with oxidative stress, systemic disease, and impaired glucose tolerance, is known to be particularly involved [1,6,11,14]. IR has further been linked to post-receptor intracellular aberrations in insulin-signaling pathways, leading to reduced phosphorylation of intracellular insulin receptor substrates [18,19]. Specifically, the major underlying factors associated with systemic manifestations of the insulin-resistant state, such as mitochondria-induced cellular stress, impaired endothelial insulin transporter, and a loss of the endothelial glycocalyx barrier protecting against oxidative damage, have been identified [1,9,20].

## 3. Risk and Contributing Factors

IR is a complex, multifactorial condition. Factors that have been implicated in the etiology of IR can be broadly divided into genetic, environmental, and lifestyle risk factors [1,2,8]. There is good evidence that even in genetically predisposed individuals, environmental and lifestyle risk factors play a significant role [9,10]. With regard to environmental factors, childhood obesity and low birth weight may contribute to an increased risk of IR in later life [8,11]. There is also a suggestion from some studies that exposures such as smoking, pollutants, certain microorganisms, and some nutritional elements may also contribute to the development of IR [8,21,22,23].

Obesity is one of the major contributing factors to the development of IR [6,24]. There is, however, some evidence to suggest that a sedentary lifestyle in the absence of severe obesity can also result in significant IR [25]. An emerging field of research indicates that dietary intake and dietary composition are critical in the etiology of IR [25]. For example, excessive fructose intake contributes to IR through hepatic de novo lipogenesis and ectopic fat storage [9,25]. There is also substantial epidemiologic evidence linking accelerated aging to increased IR [8].

## 4. Global Epidemiology of IR

**Figure 1:**
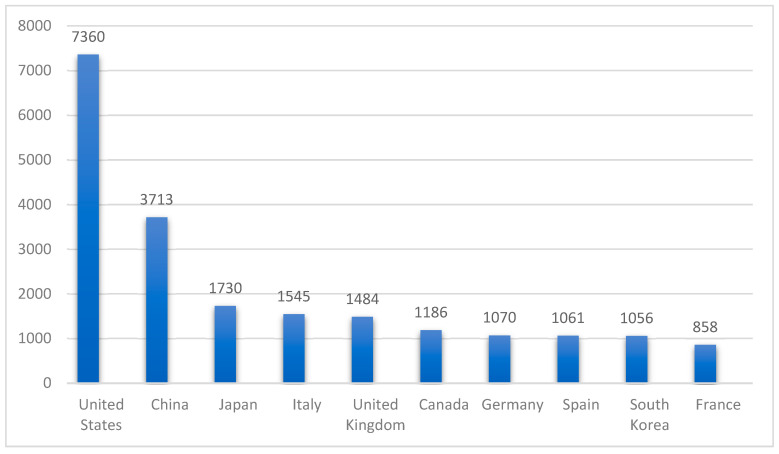
Global impact of insulin resistance research, tanking the top ten nations in terms of overall number of publications related to IR research during the period between 2002 and 2021. The numbers on the Y axis represent the number of total publications; the US is on top with 7360 publications, while France, in last place, has 858.

The global epidemic of IR is a well-known fact and there is a variation in its prevalence across different regions ranging from Europe through to South America and Asia [8,26,27]. There has been a lot of focus on the research related to IR over the last two decades and a continuous growth in the number of published articles in high-impact journals. A comparison of the 10 highest contributors is presented in Figure 1, which highlights the United States as the country with the highest number of publications. This demonstrates the perpetual increase in the public perceptions about the increasing relevance of understanding the impact of insulin resistance and metabolic health globally.

The incidence and prevalence of IR are rapidly increasing globally. The prevalence of IR in Southeast Asia from 2016 to 2021 was 44.3% [28,29]. A recent study revealed that 33.7% of the rural Brazilian population is afflicted by IR [30]. Approximately 40% of individuals in the United States aged between 18 and 40 years are impacted by IR syndrome, rendering it a reasonably prevalent illness [31]. The latest International Diabetes Federation reported that 73 million people in the Middle East have been diagnosed with diabetes, which is strongly correlated with a heightened frequency of IR [32,33,34].

In the Arab world, the rates are reported as high as the USA, although the data are not fully systematic to make any direct comparison. Nevertheless, several investigations were undertaken in Arabic nations to assess IR. A recent survey, conducted by the Fahad et al. group, assessed the rate of IR in Lebanon at 38.0% [35]. A nationwide survey in Qatar indicated that the prevalence of IR among females reached 37% [36]. Strong evidence shows that IR rates can vary within a country [37]. Populations in transition, or through urbanization, display the largest rises in IR, for example in Southeast China in comparison to rural rates and in the rapid rises in the UAE in the 1970s, most probably because of the population mobilization for oil revenue [38,39].

The persistent global nutritional shift is expected to perpetually elevate these figures; nevertheless, it remains uncertain whether IR will persist alongside diabetes and obesity or diminish at some point due to long-term dietary trends that may benefit the metabolic profile [37,38,39]. Numerous studies on various chronic diseases indicate that diabetes and IR are influenced by components of the urban obesogenic diet and lifestyle, potentially through the induction of obesity-mediated vascular inflammation [24,25,28]. Obesity is the primary factor contributing to diabetes in both industrialized and developing nations; the alteration in dietary patterns cannot surpass the significance of obesity in creating an insulin-resistant condition [10,13,37].

## 5. Prevalence of IR in Specific Populations

There is a significant variation between different ethnic groups regarding the prevalence of IR and type 2 diabetes [4,37,38]. The prevalence of IR also varies significantly depending on wage, employment status, and other indicators of socioeconomic status, with the poorest and least employed persons having the highest rates of IR [37,38,39]. Certain age groups have been shown to have a disproportionately high level of IR when compared to older or younger adults [28,39,40].

Lifestyle factors and inherited genetic risk factors could explain prevalence disparities in specific demographic groups [6,22,39]. Certain racial and ethnic groups have a higher prevalence of IR compared to the overall population [38,39]. Discrepancies exist in the literature about the level of insulin sensitivity in African or African American people [38]. In a study of 1025 participants comprising 63% non-Hispanic Whites, 9% Hispanic Whites, 11% East Asians, 11% South Asians, and 6% African Americans, it was reported that non-Hispanic Whites and African Americans displayed greater insulin sensitivity than East Asians and South Asians. Some studies have shown that surrogate estimates of insulin resistance may be higher in African Americans compared to their European counterparts [38,39]. The findings also suggest a vast proportion of Hispanic and African American people are at high risk for complications associated with IR, suggesting targeted intervention with these populations [38,39]. Further investigations are required to enhance awareness about the ethnic distribution of insulin resistance.

## 6. Molecular Mechanisms of Insulin Signaling

**Figure 2:**
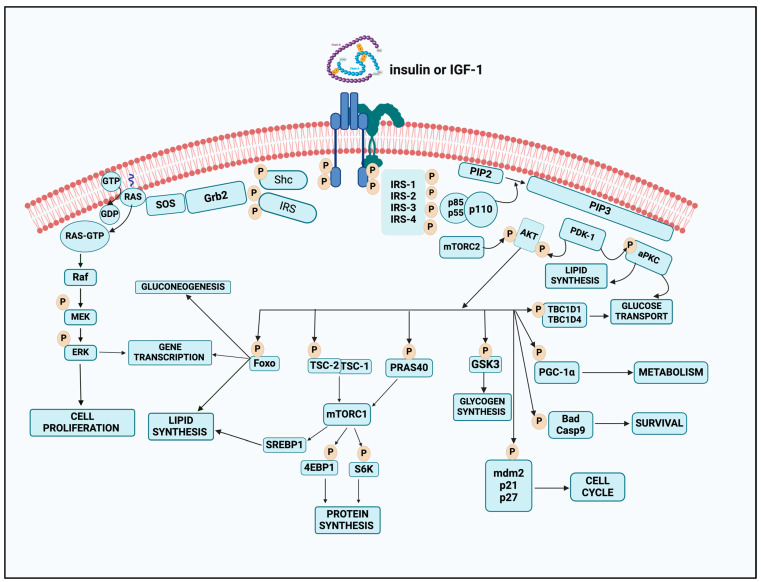
A schematic illustration of the insulin-signaling mechanism. The initiation of a chain reaction of phosphorylation events is triggered when insulin and IGF-1 receptors are activated by their respective ligand (insulin). During the process of ligand binding, the receptors undergo a conformational change and autophosphorylation. This results in the recruitment and phosphorylation of receptor substrates, such as IRS and Shc proteins. The Ras-MAPK pathway is activated by Shc, whereas the PI3K-Akt route is primarily activated by IRS proteins. This is accomplished by the recruitment and activation of PI3K, ultimately resulting in the production of the second messenger PIP3. PIP3, linked to the membrane, has the ability to recruit and activate PDK-1, which then phosphorylates and activates Akt as well as atypical PKCs. In addition to regulating glucose transport, lipid synthesis, gluconeogenesis, and glycogen synthesis, Akt is responsible for mediating the majority of insulin’s metabolic actions. Akt also regulates the cell cycle and the survival behavior of cells. The Shc-Grb2-SosRas-Raf-MAPK pathway is responsible for controlling the transcription of genes and the proliferation of cells. This image was drawn using BioRender software (https://app.biorender.com/illustrations/66fe49992ddf61bf4d5a35f6, accessed on 14 February 2025) (Science Suite Inc., Toronto, ON, Canada, DBA BioRender #2827-9028).

Insulin, a peptide hormone synthesized by the beta cells of the pancreas, is a positive regulator of numerous metabolic pathways, which, when stimulated, act to lower circulating glucose levels. Insulin and insulin-like growth factors (IGF) are involved in the process of the insulin-signaling pathway, which involves multiple stages [40,41]. The first stage involves the participation of insulin and IGF, which bind to insulin and IGF receptors, respectively. In the second step, an insulin receptor is responsible for binding to its direct substrates. These substrates include growth factor receptor-bound protein 2 (GRB2), Src homology 2 domain-containing adapter protein (SHC), insulin receptor substrate (IRS), SH2B adapter protein 2/adapter protein with a PH and SH2 domain (SH2B2/APS), and growth factor receptor-bound protein 10 (GRB10) [40,42]. Because of this interaction, metabolism and multiple cellular-signaling pathways that are involved in mitogenesis could be activated. A schematic illustration of the insulin-signaling cascade is given in Figure 2.

### 6.1. Insulin Receptor: Structure and Function

Insulin and IGF-1 exert their biological effects through the insulin receptor and the IGF-1 receptor (IGF-1R). These closely related tyrosine kinase receptors belong to a family that includes the orphan insulin receptor-related receptor (IRR) [6,19,40]. Insulin and IGF-1 primarily bind to their respective receptors, although both ligands can also interact with the alternative receptor, albeit with diminished affinity [41]. The insulin receptor, IGF-1R, and IRR are tetrameric proteins composed of two extracellular “α” subunits and two transmembrane “β” subunits linked by disulfide bonds. Both subunits are produced from a singular big precursor through proteolytic cleavage. The insulin receptor messenger RNA (mRNA) experiences alternative splicing of exon 11, resulting in two isoforms: isoform A, which excludes, and isoform B, which includes a 12 amino-acid sequence in the carboxy-terminal region of the α subunit [43,44]. IR-A is primarily expressed in fetal tissues and the brain, exhibits a greater affinity for both insulin and IGF-2, demonstrates a higher internalization rate than the type-B isoform, and is often up-regulated in cancer, while IR-B expression is most pronounced in the liver [44,45]. Heterotetramers consisting of an α/β dimer of insulin receptor and an α/β dimer of insulin-like growth factor 1 receptor (IGF-1R) can create hybrid receptor complexes that exhibit a preference for binding IGF-1 and IGF-2 rather than insulin [45,46]. Their creation seems to occur randomly in cells expressing both receptors and is contingent upon the relative expression levels of each receptor type [43,44,45]. The varied effects of insulin and IGF-1 in vivo primarily depend on hormone concentration and the relative expression levels of receptors across various tissues, rather than the ability of insulin receptor and IGF-1R to transmit distinct signaling pathways [47,48].

### 6.2. Intracellular Signaling Pathways

Insulin signaling, triggered at its receptors, exerts multifaceted effects on the metabolic processes, cell survival, and multiplication of specific cells. Insulin accomplishes its effects via insulin receptors that activate many pathways, including protein and lipid phosphorylation, modulation of transport processes, regulation of enzymatic systems, and control of transcriptional factors [40]. As the insulin binds to IR and/or IGF-1R, their alpha subunits undergo a conformational change, activating beta subunit kinase activity. Transphosphorylation of beta subunits activates the kinase, and the engagement of receptor substrates starts. Insulin receptor substrates 1-6 (IRS-1-IRS-6), which organize and mediate signaling complexes, are the best-characterized substrates [49].

IRS proteins’ amino terminus pleckstrin homology (PH) and phosphotyrosine binding (PTB) domains recruit them to the membrane and activated receptors [50]. Activated receptors phosphorylate numerous tyrosine residues to produce binding sites for intracellular SH2 domain molecules [51]. Although they share tyrosine phosphorylation patterns, these substrates have distinct in vivo activities [51]. IRS-1 KO mice had adequate glucose tolerance but delayed development and reduced insulin action, especially in muscle [52]. IRS-2 KO animals show faulty hepatic insulin signaling and growth reduction in specific neurons and islet cells, which leads to diabetes when b cells are lost [52]. IRS-1 KO preadipocytes differentiate abnormally, while IRS-2 KO preadipocytes differentiate correctly but exhibit decreased insulin-stimulated glucose transport. IRS-1 is needed for myoblast development and glucose metabolism in skeletal muscle cells; however, IRS-2 is needed for lipid metabolism and ERK activation [53].

IRS-3 and IRS-4 have narrower tissue distributions. IRS-3 is abundant in mouse adipocytes, liver, and lungs, but in humans, the gene is a pseudogene that produces no protein [53,54]. In mice, IRS3 gene disruption alone does not cause problems, whereas IRS-1 loss causes a severe adipogenesis deficiency [52,53]. IRS-4 mRNA is found in skeletal muscle, liver, heart, brain, and kidney and IRS-4 KO mice had little growth retardation and glucose intolerance [47,52,53]. It has also been reported that IRS-5 (DOK4) and IRS-6 (DOK5) have low tissue expression and poor IR substrates [51,52].

Besides IRS proteins, insulin and IGF-1 receptors can phosphorylate additional substrates [47,54,55]. IR and IGF-1R tyrosine phosphorylate Shc proteins, activating the Ras/ERK pathway. IR, IGF-1R, and other receptors bind to Grb2-associated binder (GAB) proteins [54,55]. GAB proteins, which lack a protein tyrosine phosphatase (PTP) domain, may participate in insulin/IGF-1 signaling in cells with low IRS protein levels. APS (SHB2) and Cbl, IR/IGF-1R substrates, attract CAP to the insulin-signaling complex [54,55]. The latter regulates insulin-stimulated glucose absorption [47,53]. SH2B1 directly binds to insulin receptors and IRS proteins to increase insulin sensitivity by catalyzing receptor catalysis and blocking IRS protein tyrosine dephosphorylation [47,54,55].

PI3K/Akt is the key route connecting IRS proteins to insulin metabolism. The interaction of two SH2 domains in the regulatory subunits to tyrosine-phosphorylated IRS proteins dictates the role of PI3K and its activation [47,49]. The catalytic subunit quickly phosphorylates phosphatidylinositol 4,5-bisphosphate (PIP2) to produce the linking molecule PIP3. The latter attracts Akt to the plasma membrane, where activation activates consequent signaling.

Three genes encode PI3K regulatory subunit isoforms. Pik3r1 encodes 65–75% of regulatory subunits, predominantly p85 alpha but also p55 alpha and p50 alpha. Pik3r2 encodes p85 beta and 20% of regulatory subunits. Pik3r3 encodes p55 gamma, which is identical to p55 alpha but expressed at low levels in most tissues [6,56]. Three genes produce the catalytic subunits p110a, beta, and delta. The regulatory subunit binds to a catalytic subunit to stabilize and inhibit it. The binding of the regulatory subunit to IRS protein’s phosphotyrosine residues reactivates it [47,57,58].

Mouse liver-specific p110 alpha and beta ablation causes glucose intolerance and insulin resistance [59]. Surprisingly, PI3K regulatory subunit knockouts like heterozygous p85 alpha deletion, p85 beta KO, and p50 alpha/p55 alpha double KO boost insulin sensitivity [60]. Reducing regulatory subunit concentration has been shown to increase insulin action through various ways. Catalytic subunits are usually less abundant than regulatory subunits, thus they compete with the enzymatically competent p85/p110 heterodimer for IRS protein binding. PTEN regulation is also connected to the p85 alpha monomer [47,61].

Recent studies have shown that p85 alpha binds to XBP-1 and modifies the unfolded protein response, contributing to insulin resistance [57,58]. IRS proteins recruit insulin and IGF-1-related proteins besides PI3K. According to proteomics studies of IRS-1 and IRS-2’s phosphotyrosine interactome, most interacting proteins, such as the adaptor proteins Grb2, Crk, and phosphatase SHP2, bind to both substrates. Several interaction partners bind exclusively to IRS-1 (Csk) or IRS-2 (Shc), DOCK-6, and DOCK-7 [62].

### 6.3. Role of Kinases and Phosphatases

The AGC family comprises around 60 human protein kinases that have been significantly conserved during eukaryotic evolution and can be categorized into 14 subfamilies. AGC protein kinase family members, including Akt/protein kinase B (PKB), p70 ribosomal S6 kinase (S6K), serum- and glucocorticoid-induced protein kinase (SGK), and several PKC isoforms, particularly the atypical PKCs, mediate most of the physiological effects of PI3K-generated PIP3 [63]. Serine and threonine phosphorylation activates AGC kinases, which have similar structures [64]. PDK-1 is the major upstream kinase that activates PI3K-regulated AGC kinases [63]. The PH domain of PDK-1 interacts with membrane-bound PIP3 and activates AGC protein kinases at serine/threonine residues like Akt Thr-308 [65]. Akt must be phosphorylated at Ser-473 by mTORC2 for complete activation [63,64,65]. DNAPK (DNA-dependent protein kinase) phosphorylates and activates Akt in response to DNA damage and controls insulin dependent regulation of metabolic genes such fatty acid synthase [63,66]. Different genes encode three serine/threonine protein kinases in the Akt/PKB family [65,66]. All isoforms use PH domains to attract PIP3 to the plasma membrane. Insulin affects metabolism largely through Akt2 in insulin-sensitive tissues. Unlike Akt1 and Akt3 KO mice, Akt2 KO mice develop diabetes and are insulin resistant [63].

Activation of Akt via PDK-1 and mTORC2 leads to phosphorylation and the activation of downstream targets [6,61]. TSC-2 phosphorylation by Akt degrades the tumor suppressor complex of TSC-2 and TSC-1 and activates mTORC1. The mTORC1-inhibitor PRAS40 can be activated by Akt by phosphorylating it [67,68]. The mTORC1 complex then phosphorylates and inhibits 4E-binding protein1 (4E-BP1), activates S6K1, S6K2, and SREBP1, and regulates metabolism, protein synthesis, and cell development genes [67,68]. Foxo transcription factors control lipogenic and gluconeogenic genes. Akt phosphorylates Foxos widely, giving docking sites for 14–33 family proteins. This interaction keeps Foxo out of the nucleus, limiting transcription [69,70]. Foxo1 liver ablation normalizes excessive glucose production and severe hepatic insulin resistance in animals lacking Akt1 and Akt2. This shows that insulin modulates hepatic glucose synthesis independently of the Akt/Foxo1 axis [69].

Several other Akt substrates impact insulin. TBC1D4, also known as AS160, and its homolog TBC1D1, which are implicated in insulin- and contraction-mediated glucose absorption, are phosphorylated by Akt. Inactivating and activating glycogen synthase kinase3 with Akt increase hepatic glycogen [6,71]. Akt-dependent PGC-1alpha suppresses gluconeogenesis and fatty acid oxidation [67,68]. Akt activation activates PDE3B and reduces cyclic AMP, which inhibits adipocyte lipolysis and b cell insulin release [72,73].

### 6.4. AKT Pathway of Insulin Action

Akt regulates enzymes, transcription factors, cell cycle regulators, apoptosis and survival proteins, as well as other insulin activities [74]. Murine double minute 2 (Mdm2), phosphorylated by Akt, reduces p53-mediated apoptosis and promotes cancer [75]. By phosphorylating the cell cycle inhibitors p21 IKK/WAF1 and p27Kip1, Akt causes cytoplasmic localization, cell proliferation, and apoptosis suppression [75,76]. Akt phosphorylates and inhibits Bax, Bad, and caspase-9 to increase cell survival. IKK phosphorylation by Akt promotes NF-kB [77]. Akt induced phosphorylation and activation of endothelial nitric oxide synthase (eNOS), which produces the vasodilator and anti-inflammatory molecule NO, may relate insulin resistance to cardiovascular disease [77]. Serum and glucocorticoid-induced protein family of kinases (SGKs) are very similar to Akt but rarely studied in insulin signaling. They are activated by simultaneous phosphorylation by PDK-1 and mTORC2 in a PI3K-dependent manner and share several subsequent substrates with Akt [78].

### 6.5. Interplay of PKC Isoforms

The role of PKC isoforms is well established in the regulation of metabolic influences of insulin. The PKC family has three main groups: the atypical PKCs (aPKCs), which include the ζ and ι/λ isoforms; the novel PKCs (nPKCs), which include the θ, η, ε and δ isoforms; and the classical PKCs (cPKCs), which include the α, βI, βII, and γ isoforms [79,80]. Obese and diabetic individuals have lower expression and activation of the atypical PKCs (aPKCs) [81,82] which otherwise play an important role in the insulin-dependent uptake of glucose and lipid biosynthesis. The activation of most of PKC isoforms is dependent on the phosphorylation cascade by Phosphoinositide-dependent kinase-1 (PDK-1 [83]. PKC-l and PKC-ζ facilitate insulin-dependent glucose equally. Mice with muscle-specific PKC-l deletions exhibit reduced insulin-stimulated glucose uptake I and a consequent IR [84]. In mice lacking PKC-l in the liver, insulin-induced SREBP1c expression and triglycerides are reduced, improving insulin sensitivity [85].

From this perspective, the majority of aPKC-dependent processes would be seen as “good”, as each of these metabolic processes is necessary for survival and overall health during times of sporadic and restricted food intake [84,85]. However, the remarkable effectiveness of aPKC-dependent processes in the liver that lead to excessive increases in lipogenic, glucogenic, and cytokine-producing enzymes would be conducive to the development of metabolic syndrome features, which in turn would lead to type 2 diabetes mellitus and atherosclerosis in situations where food intake is frequent and excessive. Furthermore, as is known to occur in type 2 diabetes, a concurrent deficit in aPKC activation in muscle would exacerbate the propensities for the development of metabolic and diabetic disorders [6].

### 6.6. Alternate Insulin Signaling: GRB2-SOS-RAS-MAPK Cascade

Another important insulin/IGF-1 signaling pathway branch is Grb2-SOS-Ras-MAPK, activated independently of PI3K/Akt. IRS and activated receptors bind to Grb2 and Shc adaptor molecules with SH2 domains [9]. Grb2’s amino-terminal domain binds to proline-rich proteins like Son of Sevenless (SOS), a guanine exchange factor, whereas its carboxy-terminal SH3 domain binds Gab-1. Ras-bound SOS catalyzes the transformation of membrane-bound Ras from its inactive GDP to its active GTP form. Consequently, Ras-GTP boosts downstream effectors like Raf, MEK1, and 2, which, in turn, phosphorylate and activate ERK1 and 2. ERK1/2 phosphorylation and activation of cytosol and nucleus targets affect gene expression, extra-nuclear activities, and cytoskeletal reorganization to promote cell proliferation or differentiation [6,9,85]

### 6.7. Modulation of Insulin Action

Given the magnitude of insulin and IGF-1 actions, the whole cascade needs to be tightly regulated to prevent metabolic disruptions and cancer development. Signal intensity and duration greatly affect pleiotropic effect response specificity. Turning off the insulin signal rapidly in multiple dimensions is a metabolic necessity to prevent the undesired influences. These inhibitory systems can alter the pathophysiological dynamics and inadvertently lead insulin resistance.

Cytoplasmic and transmembrane protein tyrosine phosphatases like PTP1B and LAR reduce IR, IGF-1R, and IRS activity by dephosphorylating their tyrosine residues [47,86]. The role of LAR in insulin signaling in vivo is debatable, PTP1B is crucial to insulin action. PTP1B KO mice had better insulin sensitivity, enhanced muscle and liver IR phosphorylation, and resistance to high-fat diet-induced obesity and insulin resistance [87].

PP1 (serine/threonine phosphatase protein phosphatase 1) controls glucose and lipid metabolism rate-limiting enzymes, including glycogen synthase, hormone-sensitive lipase, and acetyl CoA carboxylase [6,47]. PP2A regulates Akt, PKC, S6K, ERK, cyclin-dependent kinases, and IKK, which account for 80% of cell serine/threonine phosphatase activity [88]. Several studies show hyperactivated PP2A in diabetics [89]. There are other serine/threonine phosphatases that also have been reported to impact insulin action. Akt is dephosphorylated by calcineurin (protein phosphatase 2B or PP2B) [90]. The novel PH domain leucine-rich repeat protein phosphatases, PHLPP-1 and -2, regulate insulin action by dephosphorylating Akt and PKCs. By suppressing Akt and glycogen synthase kinase 3, PHLPP1 overexpression reduces glycogen synthesis and glucose transport [91,92]. In obese and diabetic adipose tissue and skeletal muscle, PHLPP-1 levels are increased, while Akt2 phosphorylation is decreased [91,92].

### 6.8. Role of Lipid Phosphatases

Lipid signaling is now a recognized mechanistic underpinning of insulin action. Lipid molecules from all known classes serve as signaling entities for important cellular responses. Disruptions of cellular lipid homeostasis often lead to lipid oversupply and buildup of different bioactive lipid intermediates or “lipotoxicity” in non-adipose tissues [6,93]. PIP3 levels are essential for the regulation of insulin action and are under the constant influence of phosphatases. The PTEN dephosphorylation of PIP3 impairs cell PI3K signaling [94,95]. It has been reported that insulin sensitivity rises in mice with muscle, adipose, and liver PTEN loss. Whole-body PTEN haploinsufficient mice have better glucose tolerance and insulin sensitivity [96,97]. Recent reports show that the p85alpha regulatory subunit of PI3K directly binds to and promotes PTEN activity, forming a unique PIP3 synthesis and degradation interface [58,98].

Two enzymes, SH2 domain-containing inositol 5-phosphatases (SHIP) 1 and 2, are involved in the dephosphorylation of PIP3. More efficient is SHIP2 modulation in insulin signaling, as this enzyme is more ubiquitous [99]. Its role in glucose and energy balance is reiterated by SHIP2 deficiency in mice, which causes hypoglycemia, insulin-induced Akt activation, and resistance to high-fat diet-induced obesity [100]. However, SHIP2-overexpressing animals exhibit decreased liver, fat, and skeletal muscle insulin-induced Akt activation [100,101].

### 6.9. Regulatory Roles of Grb, SOCS, Trb3 and IP7

It has been reported that Grb10 and Grb14 inhibit IR and IGF1R activity and restrict substrate availability to active receptors [102,103]. Grb10 gene-deficient mice have shown enhanced growth, insulin signaling, and glucose tolerance [104,105]. On the other hand, growth impairment, glucose intolerance, and insulin resistance result from Grb10 overexpression [47,106]. Insulin-resistant animal models and T2DM patients have elevated Grb14 expression in adipose tissue, while Grb14 KO mice had enhanced glucose tolerance and insulin sensitivity, suggesting that Grb14 suppresses insulin signaling [102,103]. Because they do not boost insulin signaling, Grb10 and Grb14 work similarly. SOCS (suppressors of cytokine signaling) adaptor proteins inhibit cytokine and growth factor signaling. SOCS proteins, especially SOCS1 and SOCS3, negatively affect insulin signaling, linking cytokine signaling to insulin resistance [107,108]. In obesity, their expression increases, and they impede the insulin receptor’s tyrosine kinase activity, compete for receptor binding, or degrade IRS proteins, causing insulin resistance [47,109].

Trb3 (Tribbles homolog 3), the expression of a pseudokinase, is upregulated in the liver during fasting and diabetic states. The resultant Trb3–Akt binding impairs insulin signaling. Trb3 knockdown increases mouse glucose tolerance [47,110,111]. Trb3 overexpression lowers insulin-stimulated S6K activation in cultured cells, while Trb3 reduction promotes it [110,111]. Trb3 apparently works independently of Akt in the adipose tissue. Trb3 ubiquitinates and destroys acetyl-CoA carboxylase to accelerate lipolysis, while insulin stimulates lipogenesis. Transgenic mice overexpressing Trb3 in adipose tissue demonstrated greater insulin sensitivity and fatty acid oxidation, protecting them from diet-induced obesity [111,112]. Inositol phosphate (IP7) is another negative regulator of insulin signaling [113,114]. Insulin and IGF-1 increase IP7, which blocks Akt translocation to the plasma membrane and activation, perhaps reducing insulin signaling [114]. The deletion of the enzyme Inositol hexakisphosphate kinase 1 (IP6K1), responsible for IP7 synthesis, boosts insulin responsiveness in mice [47,115]. More research is needed to understand these phenomena in human health and disease.

### 6.10. Role of Phosphorylation Cascade Induced Activated Serine—Threonine Kinases

**Figure 3:**
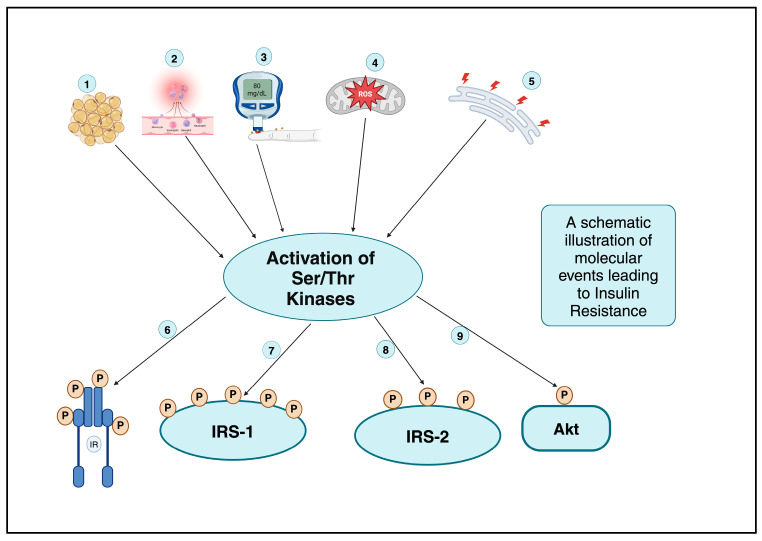
A schematic illustration of the activation of Ser/Thr kinases leading to the phosphorylation cascade on insulin receptors, insulin receptor substrates, and Akt, culminating in insulin resistance. 1—dyslipidemia; 2—inflammatory processes; 3—hyperglycemia; 4—reactive oxygen/mitochondrial stress; 5—endoplasmic reticulum stress; 6—protein kinases C and A phosphorylating insulin receptor; 7—IRS-1 phosphorylation by multiple kinases, including classical and novel PKC, JNK, IKK, S6K1, GSK3, SIK2, MAPK, and mPLK1; 8—iRS-2 phosphorylation involving JNK and GSK3; 9—Akt phosphorylation involving atypical PKC. The letter “P” in the pink circles represents phosphorylation status. This image was drawn using BioRender software (Science Suite Inc. DBA BioRender #2827-9028).

It is well established that insulin-receptor IGF-1R and IRS proteins need phosphorylation on their selective tyrosine residues for their optimum functionality in insulin signaling [47,53]. However, serine and threonine phosphorylation of the IRS complex mostly lowers insulin signaling [47,55]. These events are schematically represented in Figure 3.

Cytokines, fatty acids, hyperglycemia, mitochondrial dysfunction, endoplasmic reticulum (ER) stress, and insulin induce inhibitory Ser/Thr phosphorylation of IR, notably IRS-1 and -2, via JNK, IKK, traditional and novel PKCs, mTORC1/S6K, and MAPK [6,47,116]. Insulin-resistant rats and humans have shown enhanced IR serine phosphorylation and decreased tyrosine kinase activity equally [47,117]. As can be visualized in Figure 3, PKA-dependent inhibitory serine phosphorylation of IR occurs when cAMP levels rise [47,118]. The phosphorylation at Ser-307 is the most prevalent of serine phosphorylations, although it occurs at other sites as well [119,120]. It has been reported that obese and diabetic mice show enhanced IRS-1 Ser-307 phosphorylation rises [119,120]. This suppresses insulin receptor kinase activity, which may cause insulin resistance; however, some authors disagree with this phenomenon [47,119,120]. Human insulin can activate IRS-1 phosphorylation on Ser-307 and mice fed on a high-fat diet; an IRS-1 Ser307Ala mutant exhibited more severe insulin resistance than controls, demonstrating that Ser-307 is essential for insulin signaling. Thus, higher IRS-1 Ser-307 phosphorylation has been linked to insulin resistance, but its “cause and effect” relationship needs more investigation [47,121,122].

DAGs (diacylglycerols), a product of lipid metabolism, can activate classical and novel protein kinase C members to phosphorylate IRS proteins and IR at Thr-1336, Thr-1348, and Ser-1305/1306, impairing insulin signaling [18,40,47]. The deletion of any novel PKC family members decreases IRS-1 Ser-307 phosphorylation, reducing liver and skeletal muscle insulin resistance [47,119]. Serine phosphorylation of IRS-1 and Akt under the influence of atypical PKC-ζ inhibits the recruitment of the latter to the plasma membrane and inhibits insulin signaling [1,118,121]. mTORC1 constitutes yet another component of the negative modulator of the insulin signaling. Enabling mTOR and S6K activity increases serine phosphorylation and lowers IRS tyrosine phosphorylation, limiting insulin signaling [53,116,117]. This type of phenomenon with increased insulin sensitivity has been found in lean S6K null mice [53,116,117]. In addition, it has also been reported that Grb10 is phosphorylated and stabilized by mTORC1, blocking insulin [122,123].

## 7. Cellular and Tissue Specificity of Insulin Action

During development, the functional diversity of cells and tissues is reflected in the utilization of numerous regulatory systems, of which insulin is particularly important. This is underscored by the complexity and versatility of the mechanisms responsible for insulin action, operative in the formation of a multiplicity of fibers within a single muscle, for instance, or of diverse patterns of gene expression within the liver or adipose depots. These manifestations of cellular and tissue specialization enact modifications in the entire molecular organization responsive to insulin in conjunction with those attributable to differences in the propensity for different elements of an insulin signal transduction mechanism.

### 7.1. Adipose Tissue

The primary physiological role of insulin in white adipose tissue is to inhibit lipolysis, hence diminishing hepatic glucose production (HGP) by lowering gluconeogenic substrates [124]. The mechanism by which insulin suppresses lipolysis remains incompletely elucidated, but it is thought to be mediated by phosphodiesterase 3B (PDE3B) via diminished cyclic adenosine monophosphate (cAMP)-dependent protein kinase A (PKA) activity [6,116]. Moreover, PP1 and protein phosphatase-2A (PP2A) seem to facilitate the inhibition of PI3K-dependent insulin-induced lipolysis by dephosphorylating lipolytic regulatory proteins [116,124]. Insulin facilitates glucose transport by signaling the phosphorylation of targets related to vesicle tethering, docking, and fusion; nonetheless, its role in overall glucose disposal is relatively insignificant [125]. Insulin facilitates lipogenesis in white adipose tissue by activating SREBP-1c, signaling the translocation of glucose or fatty acid transport proteins (FATPs), enhancing fatty acid esterification, and stimulating adipogenesis via the transcription factor peroxisome proliferator-activated receptor-γ (PPARγ) [126].

### 7.2. Skeletal Muscle

Insulin signaling in skeletal muscle facilitates glucose absorption and overall glycogen production. Insulin enhances glucose transport activity through the orchestrated translocation and fusion of glucose transporter type 4 (GLUT4) storage vesicles (GSVs) with the plasma membrane in skeletal muscle [47,127]. Upon activation by insulin signaling, Akt inactivates AS160 (GTPase-activating protein [GAP] AKT substrate of 160 kDa, often referred to as TBC1D4), hence activating tiny Rab GTPase protein switches that regulate vesicle trafficking [128]. Insulin-stimulated Akt enhances the guanosine triphosphate (GTP)-bound variant of Ras-related C3 botulinum toxin substrate 1 (RAC1), facilitating GLUT4 translocation through the induction of cortical actin rearrangement [129]. Conversely, insulin regulates net glycogen synthesis in skeletal muscles by inhibiting glycogenolysis and facilitating glycogen synthesis. Insulin signaling enhances glycogen synthase (GYS) activity through the Akt-mediated phosphorylation of glycogen synthase kinase 3 (GSK3) and the activation of protein phosphatase 1 (PP1), which facilitates GYS dephosphorylation. Furthermore, insulin modulates glycogen phosphorylase activity through the dephosphorylation of phosphorylase kinase [130].

### 7.3. Hepatic Insulin Action

Insulin in the liver stimulates IRTK, which phosphorylates IRS1 and IRS2, finally activating Akt2, thus reducing HGP, promoting glycogen synthesis, and transcriptionally stimulating lipogenesis [121]. The principal role of hepatic insulin signaling is to diminish HGP by inhibiting gluconeogenesis through Akt-mediated phosphorylation of forkhead box O1 (FOXO1), which sequesters FOXO1 from the nucleus, thereby obstructing the transcriptional activation of gluconeogenic genes, including glucose-6-phosphatase (G6PC) and phosphoenolpyruvate carboxylase (PEPCK) [6,121]. Insulin not only inhibits gluconeogenic gene expression but also suppresses hepatic gluconeogenesis by restraining adipocyte lipolysis, thereby diminishing the substrates available for gluconeogenesis in the liver [121]. Moreover, in addition to inhibiting HGP, insulin enhances hepatic glycogen production via modulating GYS (particularly GYS2 in the liver) and glycogen phosphorylase via GSK3 and PP1, similarly to the process in skeletal muscles [131]. Insulin stimulates lipid anabolism by upregulating sterol regulatory element-binding protein 1c (SREBP-1c), a principal transcriptional regulator of hepatic de novo lipogenesis, thereby enhancing the transcription of various lipogenic genes, including acetyl-CoA carboxylase 1 (ACC1), fatty acid synthase (FAS), and glycerol-3-phosphate acyltransferase 1 (GPAT1) [121,132].

## 8. Insulin Resistance

The hallmark of insulin action is the homeostasis of body glucose levels, which is achieved by different and intricate networks of reactions, as discussed above. Insulin resistance is a multifaceted pathophysiological condition, characterized by diminished bodily response to insulin, leading to increased blood glucose levels, which ultimately may manifest as T2DM and metabolic syndrome [9]. In humans, IR is a significant public health issue, and its link to T2DM, metabolic syndrome, and cardiovascular disease is well-established [133,134]. In addition to the dysglycemia, it is also associated with aberrant lipid buildup and heightened lipid catabolism in adipocytes [133,134]. Insulin resistance thus fosters obesity, T2DM, and its complications, including non-alcoholic fatty liver disease (NAFLD), tumors, cardiovascular disease, and other metabolic disorders [134,135]. As a result, IR poses a significant hazard to human health and impacts quality of life. Therefore, it is imperative to understand IR comprehensively and explore innovative therapeutic approaches to mitigate the disease burden.

### 8.1. Factors Contributing to Insulin Resistance

Various factors contribute to the so-called multifactorial insulin resistance, including genetic, molecular, physiological, and metabolic pathways that result in diminished insulin action in peripheral tissues, such as muscle and adipose tissues. The development of multifactorial insulin resistance is significantly influenced by both genetic predisposition and environmental factors, which frequently interact with one another. Among several environmental factors, inappropriate nutrition, insufficient physical activity, or excessive physical activity coupled with inadequate sleep are critical. Additionally, beyond an unhealthy diet and insufficient physical activity, insulin resistance is considerably affected by factors such as endocrine dysfunctions and socio-economic conditions. These factors are discussed one by one in the following section.

#### 8.1.1. Obesity and Adipose Tissue Dysfunction

Obesity and overnutrition cause lipotoxicity in muscles, the heart, the liver, the pancreas, and other cells due to persistent FFA overproduction and dietary lipid accumulation [130,136]. It has been reported that ectopic fat deposition and fat accumulation in adipose tissues generate pro-inflammation and consequent insulin resistance [137,138]. Ectopic fat produces harmful lipids, such as ceramides and DAG, alters the PI3K pathway, upregulates PKC, JNK, and IKK complex, and results in ROS production [137,138]. In addition, the harmful effects of these fats include ER stress, membrane stiffness, inflammation, and apoptosis [137,138]. Some investigators reported no significant connection between total DAG and insulin resistance [7,139] which is an open area of further investigation. Dietary lipids activate the MAPK and NFκB signaling pathways, leading to increased NALP3 (nucleotide-binding domain, leucine-rich repeat/pyrin domain-containing-3) expression and systemic inflammation [7,140]. LPS causes inflammation by activating TLR4 and NF-κB, P38 MAPK, and other pathways [141,142]. Sun et al. (2017) reported that NOX4 (NADPH oxidase 4) mediates LPS-induced inflammation in human peripheral blood mononuclear cells [143]. LPS has also been reported to interact with mouse and human caspases and produce IL-1β after stimulating inflammasomes [144].

Holland et al. (2011) reported that TLR4 needs saturated fatty acids to cause insulin resistance, and IKKβ is crucial for TLR4-mediated pro-inflammation and ceramide production in muscles [145]. Obese mice produce and activate ceramides from absorbed and esterified fatty acids in several organs [145]. Ceramide levels rise in the skeletal muscles of obese and T2DM serum patients, whereas exercise lowers them and increases insulin sensitivity [146,147]. Ceramides activate PKCζ, which phosphorylates the PH domain of PKB/Akt on threonine residues, reducing PIP3 binding and insulin responsiveness [148]. Activating PKCζ leads to increased CD36-mediated fatty acid absorption in the liver [149]. Ceramides activate PP2A, prevent PKB/Akt translocation to the plasma membrane, and dephosphorylate Akt/PKB in differentiated adipocytes [150]. Excess FFAs increase ceramide levels, resulting in NO production in β-cells [150]. Two independent reports found that obese mice lacking Cers6 and CerS1 (ceramide modulation genes) were protected from diet-induced insulin resistance and hepatic lipid unitization separately [151,152]. Insulin resistance causes human hepatic de novo lipogenesis, which increases liver ceramide and lipid accumulation and decreases insulin sensitivity [153].

#### 8.1.2. Inflammatory Mechanisms in Insulin Resistance

Obesity leads to an appreciable rise in adipocyte size, resulting in heightened adipocyte mortality due to insufficient oxygen delivery amid the expansion of adipose tissue [154,155]. The hypertrophied adipocytes and adipose tissues subsequently generate FFAs, ROS, and pro-inflammatory adipokines [156,157].

The adipose tissues in obese individuals release many pro-inflammatory adipokines, including MCP-1, TNF-α, IL-1β and IL-6 [158]. MCP-1 recruits monocytes during chemotaxis by attracting C-C motif chemokine receptor 2 to obese adipose tissues [159,160]. As part of the inflammasome, monocytes develop into macrophages [160]. The adipose tissue macrophages metamorphose from an anti-inflammatory M2 to a pro-inflammatory M1 phenotype [161]. Resident pro-inflammatory M1 macrophages secrete cytokines, such as MCP1, IL-1β, and IL6, which may attract more monocytes, contingent upon adipocyte size and environmental factors [161,162]. The macrophages encircling the necrotic adipocytes form a crown-like structure (CLS) to phagocytize the deceased adipocytes; consequently, lipids from these adipocytes are absorbed by macrophages, disrupting their normal function. The occurrence of CLS is significantly associated with metabolic disorders and inflammation [162,163].

Increased systemic TNF-α in obesity leads to increased activity of IKK, p38 MAPK, JNK, and PKC proteins, which modify IRS protein serine residues and hinder tyrosine phosphorylation, causing insulin resistance in adipose tissues, muscles, and the liver [47,163]. PTP1B stimulation by TNF-α inhibits insulin signaling by dephosphorylating phospho-tyrosine residues in the insulin receptor and IRS protein [163,164]. Elevated IL6 can activate JAK-STAT signaling pathways and increase SOCS1 and SOCS3 protein expression, which may downregulate insulin receptor function by sterically blocking IRS protein interaction or changing kinase activity [164]. STAT3 activation by IL-6 and IL-1β leads to increased TLR-4 gene expression and NF-κB activity in hepatocytes, leading to inflammation [165]. IL-1β causes p38 MAPK activation via its receptor and inhibits insulin signaling by serine phosphorylation of IRS1/2 [7,164]. TNF-α may is believed to reduce β-cell insulin sensitivity via nitric oxide mediation, contrary to the belief that a pro-inflammatory state affects β-cell function [164,165].

#### 8.1.3. Role of Oxidative Stress

It is well documented that in human adipocytes, hepatocytes, and skeletal muscles, obesity increases ROS generation and lipid peroxidation [166]. Obesity-related mitochondrial dysfunction leads to an increase in the production of ROS, including nitric oxide [7,166].

Plasma membrane NADPH oxidase (NOX) normally generates ROS to kill microbes [167] and has many isoforms. Insulin-activated NOX4 increases H_2_O_2_ generation in healthy adipocytes, inhibiting PTP1b, promoting adipocyte development, and enhancing insulin sensitivity [166,167,168]. In obesity, NOX increases oxidative stress that inactivates metabolic enzymes, damages cellular components, and promotes lipid peroxidation [169,170].

In differentiated adipocytes, NOX4 inhibition lowers reactive oxygen species creation and MCP-1 expression, while glucose and free fatty acids increase NOX4 catalyzed ROS generation [170,171]. It has been reported that NOX4 deletion in adipocytes delays adipose tissue inflammation and insulin resistance [172]. NOX4 overexpression reported in excessive food intake reduced PTP1B inhibition and enhanced insulin resistance in adipocytes [6,7]. The levels and activities of antioxidant enzymes like SOD1, catalase, and GPX have been found to be lower in obese individuals [170,172,173]. NOX is detected in human and mouse pancreatic islets and increases oxidative stress in T2DM animals [173,174]. De Vallance et al. (2019) found that obesity-related hyperglycemia and hyperlipidemia increase ROS formation through NOX, which may reduce Akt levels and cause skeletal muscle insulin resistance [168]. NOX2 activates macrophage chemotaxis and polarization in obese rats, promoting inflammation [172]. Infiltrating macrophages, like obese adipocytes, generate ROS through NOX2 in response to elevated fatty acid and glucose levels [6,7]. After 72 h of in vitro exposure, excess FFAs are reported to increase vascular cell NOX synthesis via a PKC-dependent mechanism [167]. Fat accumulation and excess lipids increase mitochondria malfunction in obese individuals elevate ROS levels leading to metabolic dysfunction and excessive expression of pro-inflammatory markers [154,168].

Overexposure to reactive oxygen species causes oxidative stress, activating transcription factors such NF-κB, increasing systemic pro-inflammatory cytokines and insulin resistance [170,172]. Fructose metabolism also produces ROS, which accumulates citrate in the TCA (Tricarboxylic or Krebs) cycle and increases substrate availability for de novo lipogenesis, promoting lipid buildup and lipotoxicity. ROS affects oxidative phosphorylation, superoxide production by NOX, glyceraldehyde auto-oxidation, chronic inflammation, PKC activation, and hyperleptinemia [167,171]. Serine/threonine kinase cascades activated by reactive oxygen species interact with numerous insulin-signaling substrates. ROS mostly targets the insulin receptor and IRS protein family. ROS activates serine kinases, hyperphosphorylating serine/threonine residues. This reduces insulin signaling catalytic activity by inhibiting IRS-1 and IRS-2 tyrosine phosphorylation [7,9]. The serine/threonine cascade, involving IKKβ, JNK, and P38 MAPK kinases, promotes pro-inflammatory reactions in the NF-κB pathway and causes insulin resistance, as illustrated in Figure 2.

#### 8.1.4. Mitochondrial Distress

Mitochondrion creates ATP catalytically to maintain normal physiological function. Mitophagy, apoptosis, fusion, and fission let it adapt to metabolic changes. Energy production, mitochondrial integrity, and metabolic changes, such chronic mitochondrial fusion, abnormal elongation, and loss of functioning, are all affected by mitochondrial dynamics disruption [173,174]. In order to prevent anomalies, the fission mechanism divides mitochondria into two, reducing their energy production capacity and their size [175,176]. Dysfunctional mitochondrial DNA may affect energy synthesis, generate reactive oxygen species, and cause oxidative stress and mortality [172,174]. Mitophagy, a lysosome fusion that destroys damaged mitochondria, increases with mitochondrial failure [174,175]. Mitophagy reduces mitochondrial quantity, indicating lower energy expenditure, which can cause lipid buildup, lipotoxicity, and mitochondria-mediated cellular death under overnutrition.

Due to their roles in high-energy processes and excessive food and lipid overload, obesity is thought to cause mitochondrial dysfunction in adipose tissue, muscle, and liver [176]. In mice fed a high-fat diet, the expression of fusion markers mitofusin 1 and 2 (Mfn1 and Mfn2) is significantly reduced, while the fission-related protein Drp1 is elevated. In obese humans, Mfn2 is reduced, but Mfn1 was not measured [177,178]. Obese adipose tissue mitochondrial dysfunctions increase biogenesis, metabolism, respiration, and fatty acid oxidation, increasing acetyl-CoA production [179,180]. In mice, a high-fat diet or obesity increased Drp1 levels in the skeletal muscles, but Mfn1 and Mfn2 levels remained unaltered [177,178]. In obese type 2 diabetics, Mfn2 mRNA expression reduced [139,140]. Excessive uptake of free fatty acids in skeletal muscle increases β-oxidation [9]. Due to increased mitochondrial fission, obese people’s skeletal muscle mitochondria shrink in size and length, resulting in mitochondrial dysfunction and insulin resistance [178,179].

In a mouse model, mitochondrial fission increased in the liver, similar to adipose tissues and skeletal muscles of obese insulin-resistant people, while mitochondrial fusion (Mfn2) decreased [180,181,182]. Acute exposure to high glucose levels in cultured hepatocytes and myocytes increases mitochondrial fragmentation and ROS production due to Drp-1 inhibition, while mitochondrial fusion reduces ROS production [175,177]. In diet-induced obesity, increased β-oxidation and VLDL production reduce hepatic lipid buildup [183]. However, mitochondrial fission reduces mitochondrial respiratory capacity and protein expression [182,183]. Obese hepatocytes increase Ca^2+^ transport from the endoplasmic reticulum to mitochondria via mitochondria-associated ER membrane, causing Ca^2+^ overload, mitochondrial dysfunction, and ER stress signaling [184]. Obesity alters mitochondrial structure, biogenesis, lipid peroxides, and inefficient fatty acid oxidation produces DAG, acetyl CoA, and ceramides [184]. Obesity and related comorbidities caused by ROS generation beyond antioxidant defense systems, causing enhanced oxidative stress, which damages DNA, lipid membranes, proteins, and enzymes in the mitochondrial respiratory chain [184]. After mitochondrial dysfunction and oxidative stress, oxidized intracellular components, such lipids, proteins, and nuclear and mitochondrial DNA, are released as damage-associated molecular patterns (DAMPs) that cause pro-inflammatory responses [185,186].

#### 8.1.5. Lysosomal Distress

The lysosome, a vital organelle in eukaryotic cells, degrades and recycles long-lived, superfluous, or malfunctioning proteins, lipids, and organelles while generating autophagy and new ATP [187]. Stress reduction, the neutralization of ROS, and cellular homeostasis depend on autophagy [187]. High carbohydrate, fatty acid, and amino acid intake increases mTOR, which reduces autophagy [185]. In contrast, AMPK inhibits mTOR and promotes autophagy during dietary deprivation [187,188]. In obesity and overnutrition, mTOR phosphorylates ULK1 protein at Ser637 and Ser757 and Atg13 at Ser258 in the ULK1 complex, inhibiting autophagosome formation [188]. mTOR-independent pathways regulate autophagosome formation in addition to the direct mTOR pathway. Obesity and lipotoxicity raise cytosolic Ca^2+^, which stresses the endoplasmic reticulum and reduces autophagic flow, potentially preventing autophagosome-lysosome fusion [189]. Lipid buildup reduces autophagy, hydrolase activity, and lysosomal acidification, increasing the likelihood of hepatocyte dysfunction [185,189]. In diet-induced obese mice, liver-specific deletion of atg7 and TFEB genes, which are associated with autophagy, worsens steatosis, while overexpression reduces weight gain and metabolic problems [188]. This shows that autophagy enhances S-nitrosylation of lysosomal proteins, causing malfunction and autophagy impairment [189]. Defective hepatic autophagy increases misfolded or unfolded proteins and lipids, which worsens endoplasmic reticulum stress and mitochondrial dysfunction, causing systemic inflammation and insulin resistance. Autophagy suppression in adipocytes by adipose-specific atg7 deletion reduces tissue content, increases mitochondrial amount, and improves insulin sensitivity [188,189].

Enhanced autophagy reduces mitochondrial number in differentiated adipocytes; however, the exact mechanism is still unknown. Adipose-specific atg7 deletion mice have lower plasma triglycerides, cholesterol, and leptin levels and resist high-fat diet-induced obesity [188,189]. Obese people have more autophagy in their subcutaneous adipose tissue and higher systemic insulin resistance [184,189]. Mouse knockout of β-cell-specific Atg7 reduces β-cell mass, caused mild ER stress and hyperglycemia and reduced pancreatic insulin levels [190]. By mating β-cell-specific Atg7 animals with leptin-deficient mice, endoplasmic reticulum stress and diabetes are seen, highlighting the importance of autophagy in maintaining β-cell homeostasis [186]. Obesity and overeating increase lysosomal autophagy in adipose tissues but decrease it in the liver and pancreas, leaving muscle tissues unaffected. Some reports contradict autophagy and ER stress-induced β-cell apoptosis, suggesting that its significance in T2DM needs more clarity [190,191]. Animal studies suggest that high-fat diets may cause β-cell failure through endoplasmic reticulum stress, lysosomal dysfunction, and mitochondrial dysfunction after a few weeks [190,191].

#### 8.1.6. Dysfunction of Endoplasmic Reticulum

The endoplasmic reticulum (ER), another crucial organelle, is not only involved in the synthesis, processing, and transport of proteins, but it also modulates Ca^2+^ homeostasis, the synthesis of cholesterol, phospholipids, and ceramides [191,192]. Endoplasmic reticulum stress is now thought to cause obesity-related metabolic abnormalities [191,193]. Obesity and overnutrition cause ER stress and chronic inflammation in mice adipose tissue and liver due to lipid and protein buildup [191,194]. Lipopolysaccharides, glucose, and saturated fatty acids also stress differentiated primary human endoplasmic reticulum in adipocytes [195]. Saturated fatty acids and hyperglycemia cause endoplasmic reticulum stress in hepatocytes and promote lipid accumulation via the mTORC1 pathway; AMPK activation suppresses this signaling and reduces nutrient-induced hepatic lipid accumulation [195]. Palmitic acid directly causes endoplasmic reticulum (ER) stress in human and mouse myotubes and β-cells, although oleic acid reduces the effects of insulin resistance [196,197]. Another report, on the contrary, claimed that palmitic acid-induced insulin resistance is independent to muscle cell ER stress [198].

Comprehensive ER stress activation is needed to create PTP1B, which promotes insulin resistance in mice fed a high-fat diet [195]. High glucose levels in obesity, such as FFAs, negatively impact β-cell function, causing glucotoxicity, ER stress, insulin production inhibition, and irreversible β-cell death via TXNIP pathway apoptosis [199]. Chronic overnutrition and obesity increase liver lipogenesis and gluconeogenesis for energy storage, causing excessive lipid buildup [200]. Excess lipids limit protein synthesis and encourage lipid formation in the endoplasmic reticulum. High “Phosphatidylcholine/Phosphatidylethanolamine” ratio causes endoplasmic reticulum stress and releases extra liver lipids into the bloodstream, worsening hyperinsulinemia [201]. Alternatively, high cytoplasmic Ca^2+^ has also been associated with ER stress and apoptosis [201]. Saturated fatty acids increase ER membrane stiffness and activate c-Jun N-terminal kinase (JNK), which decreases ER membrane fluidity and inhibits SERCA, increasing cytosolic Ca^2+^ [202]. Li et al. claim that NF-κB activation is mediated by endoplasmic reticulum stress, which involves Ca^2+^ efflux and reactive oxygen species generation due to SERCA suppression [202]. In healthy mice, FFA increases cytosolic Ca^2+^ levels and insulin production in pancreatic β-cells [201]. Increased FFA intake causes Ca^2+^ depletion in ER of pancreatic β-cells and hepatocytes, causing ER stress [200,201,203]

Multiple pathways cause systemic pro-inflammatory responses and contribute to ER stress. To reduce stress, the ER produces PKR-like ER kinase (PERK), inositol-requiring enzyme 1 (IRE1), and activates transcription factor 6 (ATF-6) [199,203]. PERK-mediated phosphorylation of eIF2α reduces protein translation and reduces ER stress. However, it also inhibits the IκB protein, releasing NF-κB from the IKK complex and promoting pro-inflammatory protein expression [199,203]. The PERK-eIF2α-ATF5 and IRE-1 pathways promote TXNIP-NLRP3 protein synthesis, leading to IL-1β secretion [203]. IRE-1 promotes the development of IKKβ, XBP1s, and JNK proteins, leading to inflammation [203]. Additionally, the ATF-6 pathway activates pro-inflammatory responses via NF-κB and inhibits anti-inflammatory PKB/AKT. Fat buildup and consequent ER stress causes insulin resistance by releasing pro-inflammatory molecules [203].

#### 8.1.7. Genetic Factors in Insulin Resistance

Insulin resistance and metabolic disorders often manifest within families due to the interaction of environmental and genetic factors; however, the complete genetic framework of these conditions remains inadequately understood [1,2,204]. Genetic factors connected with insulin resistance can be classified into genetic defects leading to abnormal insulin structure, genetic mutations in the insulin signaling system, genetic defects related to substance metabolism, and other relevant genetic anomalies [204,205]. Mutations in particular insulin-related genes lead to variant human insulins, including Chicago insulin (F25BL*), Los Angeles insulin (F25BS), and Wakayama insulin (V3AL), which demonstrate significantly reduced insulin bioactivity and lower binding affinity to the insulin receptor, consequently impacting insulin sensitivity [2,204,205]. Uncommon mutations in insulin receptor genes lead to a reduced number of cell surface receptors and compromised insulin receptor signaling pathways, resulting in hereditary insulin resistance, which is evident in patients with genetic syndromes marked by severe insulin resistance, such as type A syndrome, leprechaunism, Rabson–Mendenhall syndrome, and Donohue syndrome [204,206]. Furthermore, since numerous molecular pathways are involved in energy homeostasis and metabolism, IR results from various mutations in multiple genes, including those linked to GLUT4, glucokinase, and PPAR nuclear receptor family, among others [1,204,206].

Modifications in lipid metabolic pathways, encompassing mutations in adipocyte-derived hormones like leptin, adiponectin, and resistin or their receptors, alterations in peroxisome proliferator-activated receptors alpha, gamma, and delta, mutations in the lipoprotein lipase gene, and other genetic variations linked to adipose tissue development can affect the advancement of glycolipid metabolism and IR [204,207]. The mutation of *AKT2/PKBb* in cultured cells may disrupt insulin signaling and diminish AKT/PKB co-expression [208]. Recent breakthroughs in high-throughput genomics have clarified the relationship between protein tyrosine phosphatase N1 (PTPN1) and IR, with this association being affected by differences in DNA sequences outside the coding region of PTPN1 [204]. Healthy carriers of the T allele of *TCF7L2* rs7903146 may exhibit increased insulin production, leading to impaired β-cell function, which is associated with an elevated risk of T2DM [204,209].

#### 8.1.8. Lifestyle and Nutritional Factors in Insulin Resistance Risk

Proactive advertising and ready availability of the energy-dense and highly processed food have changed the social landscape of eating behaviors [210]. Recreational amenities have compounded the situation by promoting physical inactivity and unhealthy eating and consequently increased rates of obesity [211]. Many communities have witnessed more than a two-fold increase in obese individuals [212].

A critical 32-year prospective dataset found that an individual’s likelihood of being obese increased by 57% if a friend became obese during a 4-year period [11,212]. Same-sex people influence each other more than opposite-sex people [11,212]. Heating and cooling systems in vehicles, residences, and workplaces ensure comfortable ambient temperatures and diminish energy consumption. Prolonged exposure to the thermo-neutral zone increases the risk of obesity [213]. Industrialization, urbanization, and rising income have replaced traditional diets with sugary, fatty, and animal-protein-rich ones [214]. Westernization in middle- and low-income countries is increasing obesity and nutritional deficits [214,215]. Glucose, sucrose, maltose, dextrose, and fructose are added to foods as added sugars, also contributing to long-term obesity [214,215]. All of these are reported to decrease the expression and the activity of GLUT 4. Elevated glucose levels instantly reduce hepatocyte insulin sensitivity and cause glucotoxicity, which can cause various clinical issues. The weight of three middle-aged cohorts who consumed sugar-sweetened beverages, potato chips, and processed and unprocessed red meats varied between 1.63 and 5.24 pounds every four years compared to those who ate vegetables, fruits, whole grains, nuts, and yogurts [215]. Childhood obesity is linked to inadequate breastfeeding, high early-calorie consumption, and sugary drinks; it also increases inflammation, leading to insulin resistance [216].

#### 8.1.9. Relationship Between Age and Insulin Resistance

The fact that body composition changes as a function of age is well known. Ectopic fat formation in the liver and skeletal muscles and visceral fat deposition in the abdomen grow with age as the overall fat mass reorganizes, leading to lipotoxicity, which has long term consequences. Numerous studies show that decreasing energy expenditure dramatically results in age-dependent fat storage. After the age 20, resting energy consumption decreases by 2–3% every decade, while skeletal muscle mass reduces by 40% between the ages of 20 and 70 [217,218]. Lower physical activity and a sedentary lifestyle reduce energy expenditure by 50% in older people [217]. Elderly people, irrespective of gender, have a higher ratio of body fat to muscle mass than young adults, even when their body fat percentage decreases [218]. These phenomena generally lead to long-term insulin resistance.

## 9. Tissue Specific Insulin Resistance

### 9.1. Role of Skeletal Muscle in Insulin Resistance

Skeletal muscle absorbs glucose, with GLUT4 playing a key role [219]. Insulin also boosts skeletal muscle free fatty acid absorption. Insulin controls glucose metabolism through complicated and highly controlled signaling cascades that influence skeletal muscle differently [1,219]. Insulin regulates systemic energy balance by facilitating skeletal muscle glycogen synthesis, glucose absorption, and lipid consumption and storage [1,219]. Skeletal muscle is a major insulin resistance site in T2DM [219,220].

Insulin resistance reduces the plasma membrane GLUT4’s translocation to skeletal muscle cells, preventing glucose transport [219,220]. The majority of insulin-mediated systemic glucose absorption occurs in skeletal muscle. Recent research links skeletal muscle insulin resistance to a shorter lifespan [219]. It has been found that mice lacking IRS1 and IRS2 in skeletal and cardiac muscles had impaired glucose uptake and shorter lifespans [220,221]. These mice lived shorter than those with defects in both IRS1 and IRS2 solely in the myocardium, despite insulin resistance in the myocardium being thought to be the cause [221]. Due to skeletal muscle 5′ adenosine monophosphate-activated protein kinase activation, mice lacking IRS1 and IRS2 in cardiac and skeletal muscles did not develop hyperinsulinemia or hyperglycemia [220]. These results show that skeletal muscle glucose homeostasis is flexible. Muscle-specific mTORC2 knockout animals had reduced glucose absorption, while mTORC1 knockout mice had muscular atrophy and a shorter lifespan. Recent research showed muscle-specific Akt deletion mice have osteosarcopenia and a shorter lifespan [221]. The data suggest that sarcopenia may regulate lifespan via skeletal muscle insulin activity. The insertion of a constitutively active Akt in skeletal muscle improved insulin signaling and increased skeletal muscle growth and lowered fat pad weight in mice [222]. Thus, selectively activating Akt in muscle tissues may help prevent skeletal muscle degeneration caused by obesity and diabetes [223]. Skeletal muscle drives whole-body glycemic regulation as the main tissue for insulin-stimulated glucose homeostasis. Muscle contraction or exercise increases insulin sensitivity in skeletal muscle [1,219].

### 9.2. Role of Liver in Insulin Resistance

The liver has a profound role in glucose and lipid metabolism systemically. Abnormal hepatic insulin action is assumed to be a very important cause of insulin resistance, in which elevated insulin levels are needed to maintain glucose homeostasis in the blood [224]. Insulin decreases glycogenic enzymes and activates glycolysis and fatty acid synthesis enzymes via Akt2, which is a downstream of insulin signaling [224,225]. Insulin reduces glycogenolysis and glycogenesis, increases glycogen synthesis, and increases glycogen and lipid storage in the liver. Recently, it was reported that liver-specific, genetically engineered mice showed impaired liver insulin activity, increased dyslipidemia, and elevated HGP [225,226].

Due to low LDLR expression, these mice, fed an atherogenic diet, developed hypercholesterolemia and atherosclerosis after 12 weeks [227]. Furthermore, deletion of IRS1 and IRS2, located downstream of insulin receptor in the liver, disrupts lipid metabolism and causes severe glucose intolerance [228]. Due to decreased hepatic Akt signaling, these animals had reduced brain insulin action and increased hepatic glucose synthesis, showing a complicated organ system where hepatic insulin resistance causes insulin resistance in other organs [227,228]. In experiments conducted on mice, it has been found that overexpression of an intrinsic liver Akt variant caused decreased blood glucose levels, elevated hepatic lipid depots, and increased levels of TG in blood [224]. This is due to insulin- promoting glycogen storage, lowering blood glucose, and stimulating hepatocyte lipogenesis. Thus, increasing liver insulin signaling may not fix glycolipid metabolism issues. The differential hepatic insulin activity suggests that, downhill from Akt intervention, it controls lipogenesis and HGP via different and independent mechanisms [224]. Insulin-resistant liver pathophysiology cannot be fully explained by directly evaluating this model in mice in view of the fact that insulin regulates hepatic glucose homeostasis through direct and indirect processes [224,226,228]. More studies are warranted to fully understand the role of the liver in insulin resistance and its consequences.

### 9.3. Role of Adipose Tissue in Insulin Resistance

In addition to adipose tissue being crucial for energy storage, it is an endocrine organ that releases hormones and cytokines, such as adiponectin, leptin, TNF-α, and many chemokines [229]. Insulin sensitivity requires these molecules in adipose and other tissues. Recent studies show that adipose tissue macrophages express IL-10 during feeding and complement the insulin action to suppress HGP, helping us comprehend that many organs are affected by adipose tissue [229]. Adipose tissue needs insulin to absorb glucose and prevent lipolysis; as a result, its expansion reduces systemic insulin resistance by preventing extra lipids from accumulating in muscles, the liver, and the pancreas [229]. We understand that obesity, inflammation, and insulin resistance in adipose tissue, which contribute to T2DM, are worsened by overnutrition [229,230]. The reduction in adipose tissue insulin sensitivity causes lipolysis and the release of free fatty acids, which increases ectopic fat deposition in the liver and skeletal muscle [230,231]. Obesity-related metabolic disorders are exacerbated by excess adipose tissue infiltrating other organs, reducing insulin effectiveness [230,231]. This necessitates the need to understand the genesis of insulin resistance in adipose tissue to formulate treatment modalities of T2DM. Insulin receptor knockout studies adipocyte have reported conflicting results. Adipocyte-specific PTEN knockout mice had increased Akt signaling in adipose tissue, decreased hepatic fat accumulation, and improved obesity-related metabolic disorders [229]. Increasing insulin signaling in adipocytes allowed healthy adipose tissue expansion without inflammation or fibrosis. This shows that increasing insulin signaling in the adipose tissue may be a viable therapeutic strategy for metabolic diseases associated with obesity.

### 9.4. Role of Myocardial Tissue in Insulin Resistance

Research on insulin signaling has predominantly concentrated on cells and tissues that regulate systemic metabolic homeostasis, including the adipose tissue, liver, skeletal muscle, and brain. Nevertheless, insulin sensitivity is equally crucial in other organs, including the heart. Effective insulin signaling in the cardiac muscle regulates various cellular activities, such as cell proliferation, cell survival, apoptosis, and diverse metabolic functions [7,219,232]. Effective insulin signaling has a significant role in the Akt-mediated regulation of cardiac development, as demonstrated by the decreased heart size in insulin receptor KO mice as compared to normal mice [232]. A downregulation of genes associated with the electron transport chain was also reported in these insulin receptor knockout mice thereby interfering with the cardiac metabolism and energy outputs and reiterating the profound influence of insulin signaling in cardiomyocytes [232].

Cardiomyocytes demonstrate significant IR expression, while the insulin-like growth factor receptor-1 (IGF1R) displays comparable levels, indicating a substantial overlap in their pathways within the myocardium [232]. In cardiomyocytes exhibiting insulin resistance, insulin receptor signaling is partly complemented by insulin-like growth factor 1 receptor signaling. The interplay between IR and IGF-1R signaling is crucial for maintaining normal cardiac metabolism and function [232]. The presence of a permanently active form of Akt in the myocardium enhances insulin signaling, and it has been reported to lead to the moderate preservation of the systolic function, along with cardiovascular protection [7,232]. Addressing Akt activation in the myocardium may serve as an effective therapeutic approach for treating heart failure associated with metabolic diseases. Further research is needed to explore these possibilities in the near future.

### 9.5. Role of Other Cell Types and Tissues in Insulin Resistance

#### 9.5.1. Hypothalamic Neurons

It is well established that Insulin inhibits hunger by activating hypothalamic neurons; it also suppresses HGP via potassium-sensitive ATP channels and stimulates the growth of adipose tissue through sympathetic actions [7,233]. Given the immense role of hypothalamus in energy homeostasis, its Agouti-related protein (AgRP) and pro-opiomelanocortin (POMC) neurons play a crucial role in feeding behavior [233,234]. Mice deficient in insulin receptors in AgRP neurons IR knockout exhibited diminished insulin action in inhibiting HGP, while POMC IR knockout mice demonstrated reduced insulin efficacy in suppressing lipolysis [235]. Insulin signaling is shown to regulate glucose metabolism in AgRP neurons, while in POMC neurons, it influences adipose tissue lipolysis and improves hepatic steatosis [235]. Insulin resistance in the hypothalamus contributes to disruptions in glycolipid metabolism and diminished regulation of hunger. As a result, targeting insulin actions, particularly in the hypothalamus, seems to be a viable therapeutic method for treating obesity-related disorders, such as irregularities in hunger phenomena, increased HGP, and dyslipidemia.

#### 9.5.2. Pancreatic β Cells

Insulin is released by the pancreatic β-cells to regulate blood glucose levels and maintain metabolic balance. Overexpression of IRs in pancreatic β-cells greatly boosts insulin gene transcription and content. In mice with β-cell-specific KO of IRS2, glucose load significantly reduced β-cell mass and insulin production [236]. The activation of constitutive Akt1 leads to β-cell proliferation and hypertrophy [7,237].

A recent report that used an *Ins1*^cre^ knock-in allele to delete *Insr,* specifically in β-cells of both female and male mice, indicates that β-cell insulin resistance, characterized by decreased β-cell *Insr*, plays a role in hyperinsulinemia during glucose stimulation, thus enhancing glucose homeostasis regardless of gender and fed state in mice [237]. To treat obesity-induced diabetes and minimize β-cell mortality, specifically increasing insulin signaling may be effective treatment modality. More research is needed to comprehend how β-cell insulin signaling impacts diabetes in bigger sample sizes.

#### 9.5.3. Vascular Endothelial Cells

In vascular endothelial cells (VECs), insulin activates eNOS, lowering VCAM-1 expression and alleviating atherosclerotic events [7,238]. VEC-specific IR KO mice prevent nitric oxide induced vasodilation, increase VCAM-1-dependent leukocyte adhesion, and impair insulin’s anti-atherosclerotic activity [239,240]. Vascular endothelial cell-specific dominant-negative IR transgenic mice have reduced vasorelaxation [240]. In addition, genetic deletion of Akt1, a downstream target of insulin signaling, lowered VEC eNOS phosphorylation. In contrast, mice overexpressing IRS1, specifically in VECs, increased insulin signaling and reduced atherosclerosis [239]. The involvement of FoxOs downstream of insulin signaling in VECs is further studied. Atherosclerosis in low-density lipoprotein receptor KO mice caused insulin resistance in major arteries and decreased FoxO1 and FoxO3a phosphorylation, showing that FoxOs are activated in atherosclerotic vasculature [239]. Vascular endothelial cell-specific deletion of all three FoxOs (FoxO1, FoxO3a, and FoxO4) increased eNOS-derived NO production and decreased iNOS expression in LDLR KO mice’s VECs, suppressing atherosclerosis development [239,240]. This shows how FoxOs integrate many atherosclerosis pathways and may be therapeutic targets. These findings show that insulin signaling in VECs inhibits atherosclerosis and that insulin resistance increases atherosclerotic plaques and long-term complications.

#### 9.5.4. Macrophages

Insulin-resistant human monocytes displayed lower IR tyrosine kinase activity, and the monocyte/macrophage insulin resistance theory is gaining momentum [8,241]. In obese mice, poor insulin signaling in intraperitoneal macrophages increased CD36 expression and oxidized LDL binding and absorption, accelerating atherosclerosis [242]. Mice lacking IR in macrophages due to bone marrow transplantation had increased cholesterol absorption in aortic plaque lesions, which worsened atherosclerosis [241]. One study employing macrophage-specific IR KO mice demonstrated that insulin resistance in macrophages reduces atherosclerosis which is in variance with other reports [241]. In obese and atherosclerotic mice, insulin resistance activates macrophage transcription factors, FoxOs [8,241,242]. Myeloid-specific FoxO KO (FoxO1, FoxO3a, and FoxO4), which was elevated in atherosclerotic mice’ macrophages, surprisingly worsened atherosclerosis by stimulating bone marrow cell growth [241]. Thus, FoxOs are activated in macrophages and VECs in an insulin-resistant animal model, but their pathophysiological role in atherosclerosis differs. It is now well known that diminished insulin signaling in macrophages may cause atherosclerosis.

## 10. Consequences of Insulin Resistance

**Figure 4:**
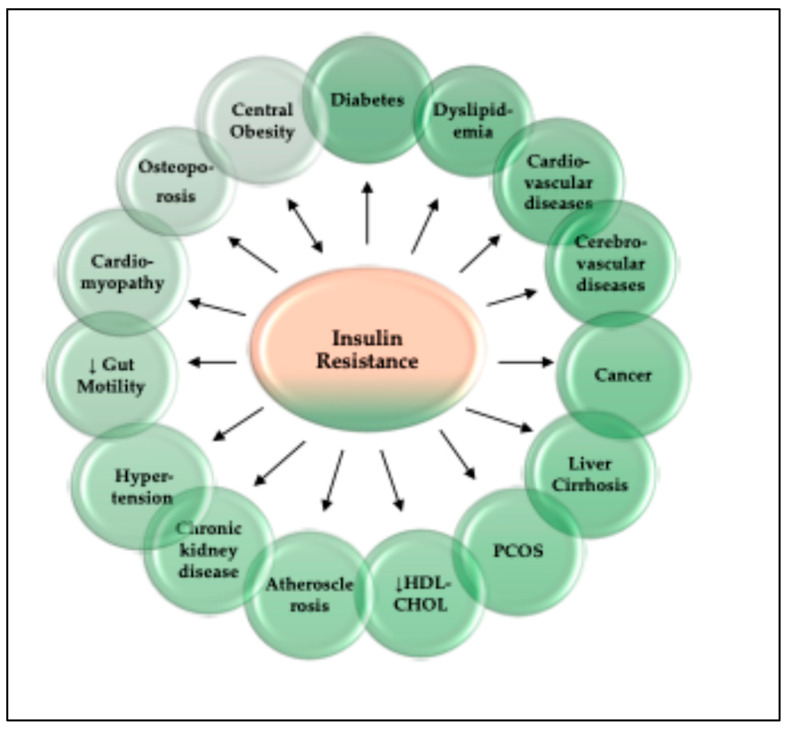
A pictorial representation of the consequences of insulin resistance.

Alterations in insulin signaling lead to a repertoire of metabolic and associated diseases, which are summarized in Figure 4 and briefly discussed as follows.

IR is related to diabetes and its associated complications [243,244]. Diabetes will affect 643 million people worldwide by 2030 and 783 million by 2045, assuming epidemic proportions that underscore the importance of this association [243,244]. Insulin resistance has a profound influence on lipid metabolism and contributes to dyslipidemia, which is an abnormal lipid profile characterized by elevated triglycerides, low HDL cholesterol, and often increased small, dense LDL particles. The alterations in the lipid metabolism in IR include increased influx of free fatty acids (FFA) into circulation. The liver uses the excess FFAs to synthesize triglycerides, which are then packaged into VLDL particles and secreted into the bloodstream. Elevated VLDL levels contribute to hypertriglyceridemia (high triglycerides), a hallmark of dyslipidemia in IR [9,245]. Lipoprotein lipase (LPL) levels are reduced, impairing the clearance of triglyceride-rich lipoproteins (VLDL and chylomicrons) from the bloodstream. This further exacerbates hypertriglyceridemia [245,246]. HDL cholesterol levels are often low in insulin resistance. This is because HDL particles are remodeled and cleared more rapidly in the presence of high triglycerides. Insulin resistance also promotes the formation of small, dense LDL particles, which are more atherogenic (more likely to contribute to plaque formation in arteries). This occurs because triglyceride-rich VLDL particles exchange triglycerides with LDL particles via CETP (cholesteryl ester transfer protein), leading to the formation of smaller, denser LDL particles [245,246]. Additionally, insulin resistance disrupts the regulation of key enzymes involved in lipid metabolism, such as acetyl-CoA carboxylase and fatty acid synthase, further promoting dyslipidemia [9,245].

Insulin resistance is considered a significant risk factor for cardiovascular diseases across various populations, including those with euglycemia and those with diabetes [247]. Mathematical modeling suggests that IR accounts for approximately 42% of myocardial infarctions, making it the most significant single cause of coronary artery disease [247]. It is reported patients with elevated HOMA-IR (homeostatic model assessment for insulin resistance) values (≥4.14) exhibited significantly reduced global longitudinal strain (GLS), increased vascular stiffness, and heightened pulse wave velocity (PWV) in the carotid artery, compared to those with lower HOMA-IR values [10,248]. Hyperinsulinemia causes diabetic cardiomyopathy through impaired insulin signaling, cardiac mitochondrial dysfunction, endoplasmic reticulum stress, autophagy, calcium handling, abnormal coronary microcirculation, neurohumoral activation, and maladaptive immune responses [246,247,248,249].

Insulin resistance has been linked io ischemic cerebrovascular illness in a data analysis spanning from 1999 to 2022 [250]. In two studies on Korean and Japanese subjects, it was reported that insulin resistance is an independent risk factor for silent lacunar infarct (SLI) and is positively linked with its occurrence and severity [251,252]. A recent meta-analysis of 11 cohort studies found a positive connection between the Ty-G index and ischemic stroke risk [253].

Insulin resistance has been identified as the primary predictor of non-alcoholic fatty liver disease (NAFLD) in both obese and lean individuals. Research indicates a strong correlation between serum insulin levels and hepatic lobular inflammation, as well as histological progression, including ballooning [254]. In patients with NAFLD, there was a significant increase in glycerol appearance and lipid oxidation, alongside an elevation in insulin resistance corresponding to the degree of steatosis [254].

Polycystic ovarian syndrome (PCOS), which affects 6–7% of the global population, has been linked with IR [255]. Excess insulin secretion activates pituitary gland insulin receptors, stimulating androgen secretion from the ovaries and the adrenal glands via the pituitary–ovary and adrenal axes and increasing free testosterone by suppressing SHBG synthesis [255,256].

Recent experimental, epidemiological, and clinical evidence indicates a synergistic relationship between IR and compensatory hyperinsulinemia in the development and progression of various cancers, including breast, colorectal, prostate, pancreatic, adrenocortical, and endometrial cancers [9,257,258]. The mechanisms underlying the association between IR and tumors remain unclear; however, they may involve multiple pathways and are likely to differ across various cancer types. IR-related factors, such as chronic persistent hyperinsulinemia, INSRs, IGF1Rs, INSR/IGF1R hybrids, chronic inflammation, ncRNAs, and microbiota, have been proposed as influential elements in all stages of tumor development [9,259,260]. The mitogen-activated protein kinase (MAPK) insulin pathway underlies numerous obesity-related malignancies that regulate cell growth and mitosis. Insulin directly facilitates cell proliferation and survival through the phosphatidylinositol-3-kinase/protein kinase B (PI3K/Akt) and Ras/MAPK pathways [260,261].

IR is a significant risk factor for the decline of renal function in non-diabetic chronic kidney disease and hypertension [262]. Insulin resistance has been linked to liver cirrhosis [263]. It has also been reported that insulin resistance may influence the relationship between insulinemia and bone mass and lead to risk of osteoporosis when HOMA-β ≥ 100 and HOMA-IR ≥ 2 [264]. Insulin resistance has also been associated with other conditions, such as postburn trauma, post-adolescent acne, and gastroesophageal reflux disease [9,244,265].

## 11. Therapeutic Modalities Targeting Insulin Resistance

The defining characteristic of insulin resistance is hyperglycemia, which is thought to result from obesity, chronic inflammation, genetic susceptibility, and ectopic fat accumulation. Thus, treatment strategies frequently seek to adjust these fundamental underlying causes. An integrated and multipronged strategy is essential to attain the optimal objectives.

### 11.1. Lifestyle Modifications

A healthy lifestyle includes exercise, which improves health and lowers IR in obese children and adolescents. The current literature suggests that aerobic, resistance, and combination training reduce IR in obese children and adolescents. Although it is uncertain what sort of exercise is best, aerobics and mixed training tend to improve IR more than resistance training.

“Exercise snacks” are short-durations frequent bouts of physical activity that provide an efficient way to help sedentary populations, encourage exercise regimens, and raise awareness of the health benefits of exercise [266]. Sedentary people can increase their cardiovascular fitness, metabolic capacity, and muscular function with exercise snacks, which are faster and easier than traditional exercise. The short duration and high intensity of exercise snacks allow for the rapid mobilization of various organ systems, which improves skeletal muscle oxygen and glucose utilization, muscle protein synthesis, and other musculoskeletal functions [266]. However, many issues remain unresolved about the benefits of various modes of exercise and need further research.

Diet is a crucial way to treat IR in children. Balanced normocaloric or hypocaloric diets work depending on the child’s age and intervention stage. The long-term effects of diets’ macronutrient contents on cardiometabolic risk, including IR, have not been studied. A low-carbohydrate diet may treat IR in children and adolescents, but more research is needed before it can be recommended. In dietary intervention techniques for children with metabolic disorders, carbohydrate quality is becoming important. The awareness about glycemic index of different diets should be increased and patients should be taught how meals affect glucose metabolic parameters [267]. They should also follow the Mediterranean diet, which may alleviate obesity and related conditions. Finally, insulin index diet research may help create new dietary therapies for obese adolescents with IR, but clinical trials are needed to prove this. To determine how nutritional supplementation and microbiome-based therapies affect IR, prospective randomized studies with extended monitoring periods are needed.

### 11.2. Pharmacologic Interventions

**Figure 5:**
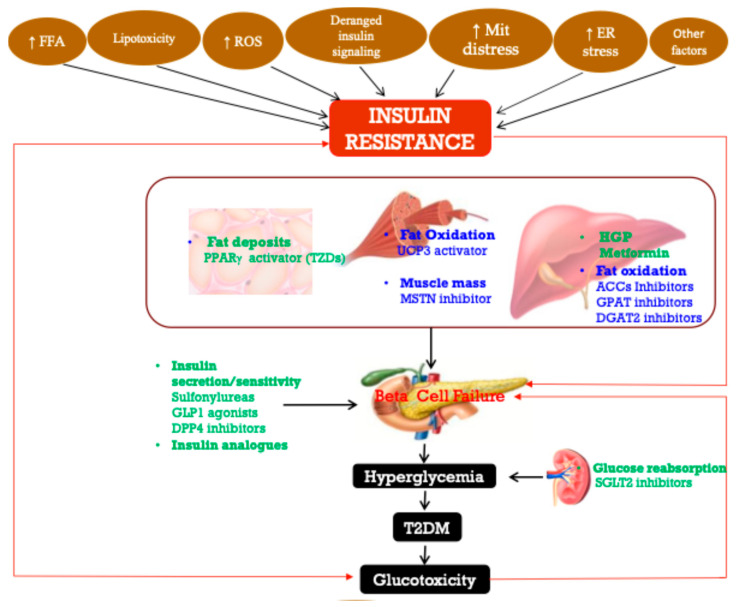
Graphic illustration of therapeutic strategies targeting insulin resistance and T2DM. The currently used modalities are shown in green. The future therapeutic options are summarized in blue. Drugs like sulfonylureas, glucagon-like peptide 1 (GLP-1) agonists, and dipeptidyl peptide-4 (DPP-4) inhibitors augment insulin secretion. Thiazolidinediones (TZDs) and metformin are insulin-sensitizing agents, targeting fat storage capacity of adipose tissue and HGP (hepatic glucose production). The potential agents targeting enhancement of ß oxidation in liver and skeletal muscle and stimulation of muscle quality. Mit—Mitochondria; ER—Endoplasmic reticulum; FFA—free fatty acid; ACC—Acetyl-CoA carboxylase; GPAT-Glycerol-3-phosphate acyltransferase; DGAT2—diacylglycerol acyl transferase 2; UCP3—Uncoupling proetein3; MSTN—Myostatin; PPARγ—Peroxisome proliferator-activated receptor-γ; SGLT2—Sodium glucose cotransporter2.

In alignment with the multifaceted etiology of insulin resistance, various therapeutic modalities are available, which directly or indirectly address distinct aspects of insulin resistance. Figure 5 summarizes the therapeutic modalities targeting IR and T2DM, and the same topic is discussed briefly in the forthcoming sections.

#### 11.2.1. Currently Used Medications

The currently used pharmacological agents alleviating the effects of insulin resistance include biguanides, thiazolidinediones, sodium-glucose cotransporter inhibitors, glucagon-like peptide-1 receptor agonists, dipeptidyl peptidase-4 inhibitors and sulfonylureas. Their brief mechanism of action and other details are explained in Table 1.

#### 11.2.2. Recent Drug Targets for Insulin Resistance

Recently developed drug targets include some pharmacologic agents that act as agonists for many signal transducers/receptors and mediators of metabolic signals. These drug targets have been developed in a series of rigorous molecular and clinical studies. A brief description of these recent drug targets is presented in Table 2.

#### 11.2.3. Future Insulin Resistance Drug Targets

**Figure 6:**
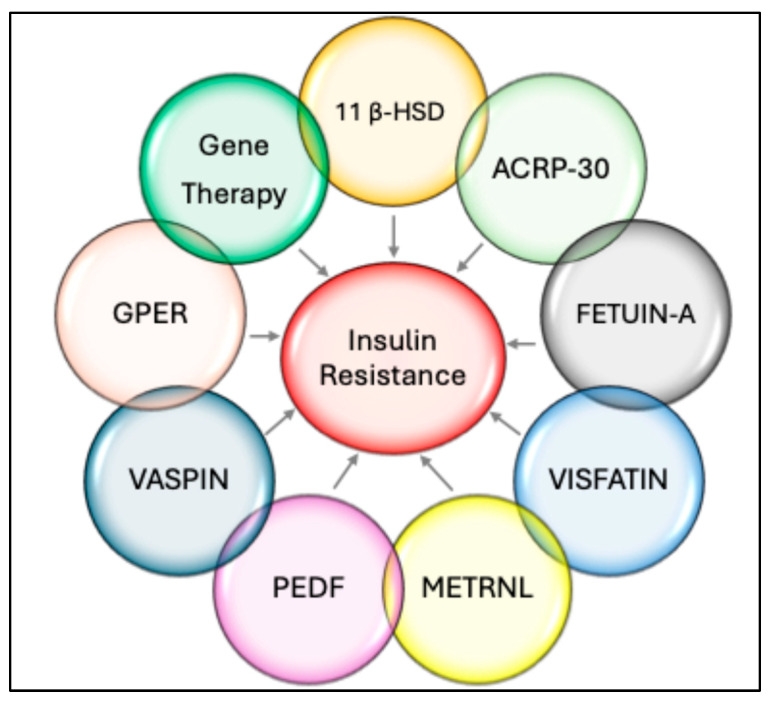
A schematic view of future drug targets aiming at insulin resistance: 11β-DH—1β Hydroxysteroid dehydrogenase; PEDF—Pigment epithelium-derived factor); GPER—(G protein-coupled estrogen receptor); METRNL—Meteorin-like.

Future targets against IR are possible molecular receptors or sites that could be used to develop new lead molecules for the treatment of IR in general and T2DM in particular. Although not much is currently known about their involvement in diabetes, these targets may be extremely important in the management of the disease. By offering safe, efficient therapy without sacrificing patient compliance, future targets provide a viable way to overcome the drawbacks of traditional and existing methods [281]. A brief description of potential future targets is schematically presented in Figure 6 and is discussed below.

##### 11β—Hydroxysteroid Dehydrogenase (11β-HSD)

This enzyme transforms the glucocorticoid, cortisone, into its active form, cortisol. 11-hydroxysteroid dehydrogenase type 1 (11β-HSD1) and 11β-hydroxysteroid dehydrogenase type 2 (11β-HSD2) are the two isoforms that are currently accessible [282]. Maintaining the levels of the 11β-HSD1 enzyme naturally enhances insulin sensitivity because it is well established that high blood glucocorticoid levels can lead to glucose intolerance. According to research published in 11β-HSD1 knockout mice, there was a reduction in blood glucose levels, enhancement in insulin sensitivity, improvement in glucose tolerance, and a lack of in vivo glucocorticoid biosynthesis [282]. Thus, by controlling the insulin-signaling transduction system, blocking 11β-HSD1 may help lower insulin resistance and thereby raise insulin sensitivity. Considering all that has been reported so far, 11β-HSD1 represents a unique molecular target for targeting IR and for the treatment of T2DM [282].

##### ACRP-30 (Adiponectin)

The adipose tissue is well-established for its capacity to store fats; however, recent studies indicate that it also functions as a source of hormones, such as resistin, adipsin, leptin, TNF-α, adiponectin, and ACRP30 [283,284]. Research indicates that serum protein ACRP-30 plays a significant role in the regulation of diabetes mellitus, while TNF-α is a principal pro-inflammatory mediator implicated in insulin resistance. Studies reveal that Acrp30 levels are diminished in various obesity and diabetes models due to elevated TNF-α levels, indicating a negative correlation between this protein and diabetes [283,284]. Additionally, mice deficient in Acrp30 exhibit insulin resistance, contributing to the onset of diabetes mellitus. Increasing circulating levels of Acrp30 may enhance insulin sensitivity and facilitate the management of blood glucose levels, positioning Acrp30 as a promising therapeutic target for diabetes mellitus treatment [284].

##### Fetuin-A

Synthesized predominantly in the liver and released into the bloodstream, Fetuin-A is the principal protein necessary for transporting FFA into circulation and is implicated in the inflammation of β-cells, which can result in β-cell degeneration in the pancreas, hence contributing to insulin resistance and many metabolic diseases [285]. Fetuin-A, in conjunction with insulin, is a significant protein that can attach to the external area of the insulin receptor. Fetuin-A inhibits the autophosphorylation of the tyrosine kinase, a major enzyme in insulin signaling, which is entirely contrary to insulin’s effect [285].

The primary interaction between insulin and tyrosine kinase regulates blood glucose levels; however, an increase in Fetuin-A concentration in the bloodstream may lead to insulin resistance and, eventually, T2DM [285]. Research indicated an enhancement in insulin sensitivity in mice possessing Fetuin-A deletion genes, demonstrating a negative correlation between Fetuin-A and insulin sensitivity in diabetes. These criteria suggest that Fetuin-A has the potential to serve as an innovative target for the management of T2DM in the near future [286].

##### Visfatin/NAMPT (Nicotinamide Phosphoribosyl Transferase)

Visfatin is a protein that has multiple functions and is also referred to as nicotinamide phosphoribosyl transferase. It was first discovered in the year 2005 in the visceral adipose tissue; however, ever since, it has been found in a variety of other organs and tissues as well. In the past, it was also known as the Pre-B colony Enhancing Factor (PBEF). It possesses insulin-like properties, which means that it helps regain insulin sensitivity. Research has demonstrated that the concentration of visfatin in the serum rises in tandem with the progression of T2DM, thereby establishing a connection between visfatin and T2DM [287]. It has been demonstrated in recent research that visfatin binds to the insulin receptor in a region distinct from where insulin does [287]. This indicates that visfatin has an activity that is like that of insulin and that it promotes cell proliferation. The exact role of visfatin in the therapeutic interventions targeting IR and T2DM is under active research.

##### Metrnl

Metrnl is an adipokine sourced from adipose tissues, predominantly found in subcutaneous white fat, and is crucial for the regulation of glucose homeostasis. The Metrnl gene, located on mouse chromosome 11 and human chromosome 17 (17q25.3), encodes this protein. It exhibits 40% homology with the neurotrophic factor Meteorin and was, therefore, initially designated as Meteorin-like [288]. Metrnl is significant in regulating energy metabolism, lipid metabolism, cardiovascular function, immunological inflammation, and insulin sensitivity [288]. Researchers found that it operates through the upregulation of the PPARγ pathway, resulting in increased insulin sensitivity in a mouse model [288,289]. It is concurrently observed that it promotes adipose tissue browning, resulting in increased energy expenditure and improved glucose tolerance.

Metrnl participates in multiple pharmacological pathways via intracellular signaling among cells. In nerve cells, it facilitates neurite outgrowth through the JAK-STAT3 and MEK-ERK signaling pathways. In adipocytes, the upregulation of Metrnl enhances lipid metabolism, mitigates inflammation induced by a high-fat diet, and promotes adipose remodeling via the upregulation of PPARγ, thereby improving insulin resistance [281,289]. In muscle cells, or myocytes, there is an enhancement in PPARγ signaling, which leads to an increased phosphorylation of AMPK due to elevated intracellular calcium levels. This process also facilitates the phosphorylation of TBC1D1, HDAC5, and p38 MAPK through an AMPK-mediated mechanism. Consequently, this promotes the expression and translocation of GLUT4, thereby improving insulin sensitivity and reducing inflammation [288,289]. Given its role in the multiple metabolic pathways, it is a promising target directed at insulin resistance and metabolic syndrome [281].

##### PEDF (Pigment Epithelium-Derived Factor)

PEDF, a glycoprotein that is secreted from human retinal pigment cells and adipose tissue is a member of the serine protease inhibitor family. It facilitates the breakdown of triglycerides into glycerol and free fatty acids, which then leads to the transfer of the free fatty acids into the systemic circulation, which is believed to lead to inflammation of the cells [290,291]. Because of the initiation of the kinase-mediated Serine/Threonine phosphorylation cascade of IRS (insulin receptor substrate), insulin signaling is reduced, which leads to insulin resistance in the cells of the body. In addition, it promotes the release of inflammatory mediators, including TNF-α and iIL-1, which ultimately results in insulin insensitivity in the body [290]. The administration of PEDF caused a decrease in insulin sensitivity, which was then restored after the administration of anti-PEDF, according to the findings of a study that investigated the effect of PEDF administration in animals [291]. PEDF has been shown to have a positive connection with insulin resistance in both children and adults [290,291]. A decrease in the levels of PEDF that are found in circulation has the potential to improve insulin sensitivity, which would position PEDF as a possible novel therapeutic target for diabetes mellitus and other metabolic disorders, respectively [281].

##### Vaspin (Serpin A12)

Vaspin, also known as Serpin A12, is a glycoprotein found in serum that belongs to the protein family known as serpins. Produced by adipocytes, it is reported to modulate insulin action [292,293]. It has been demonstrated through research that the serum levels of vaspin begin to decrease with increasing severity of diabetes [292,293]. This observation led to the belief that increasing the vaspin levels would be beneficial in the management of diabetes mellitus. In addition, it has been shown that the administration of vaspin in experimental mice results in an improvement in insulin sensitivity as well as an increase in glucose tolerance [293]. As a result of these pieces of evidence, it has the potential to be a target for the treatment of metabolic aberrations, such as obesity and T2DM [292,293]. By inhibiting the KLK7 (kallikrein 7) enzyme, which is an insulin-degrading enzyme that degrades insulin and decreases the insulin half-life, vaspin is able to accomplish its function and achieve its desired effect. The inhibition of KLK7 results in an improvement in insulin signaling, as well as an increase in the half-life of insulin, which contributes to a reduction in the levels of glucose in the blood [293]. It also performs some other actions that indirectly reduce the blood glucose from the body, such as reducing the amount of food that is consumed, which ultimately reduces the amount of glucose that is produced by the liver HGP through the hepatic branch of the vagus nerve. This is accomplished by decreasing the accumulation of soluble lipids in the liver and increasing the amount of insulin signaling in the liver. In white adipose tissue and brown adipose tissue, it decreases inflammation and boosts insulin signaling. In the central nervous system, it reduces food intake by activating the vagus nerve, which is responsible for the regulation of appetite [281].

##### G Protein-Coupled Estrogen Receptor (GPER)

GPER, alternatively referred to as G protein-coupled receptor 30 (GPR30), is a protein encoded by the GPER gene in humans. GPER interacts with and is stimulated by the female sex hormone estradiol, facilitating certain fast cellular actions of estradiol. It is an orphan seven-transmembrane G-protein-coupled receptor and is implicated in estrogen signaling [294]. Integral to the cellular membranes, it plays an important role in controlling hyperglycemia and cellular proliferation among its vast array of functions [294,295]. A GPER-deficient female mice model exhibited inadequate insulin levels, a situation that aggravates the glucose homeostasis and induces T2DM [281]. It is well established that premenopausal women have higher levels of estrogens, which is beneficial in the control of blood pressure, lipid metabolism, glucose balance, and mitigating inflammation. Subsequent to menopause, women’s susceptibility to insulin resistance and other metabolic problems increases as a result of altered estrogen levels. The role of GPER becomes essential under such circumstances, and it has been reported that a selective agonism of GPER by the agonist G-1 can mitigate obesity and metabolic dysfunction symptoms in many murine models, thereby curtailing weight gain, diminishing insulin resistance and inflammation, and enhancing glucose and lipid homeostasis in vivo [294]. Consequently, GPER constitutes a groundbreaking therapeutic target, with G-1 serving as a first-in-class therapeutic drug for the treatment of obesity and its related comorbidities, such as T2DM [281,296].

##### Gene Therapy

Gene therapy involves the modification of a gene of interest to treat or cure diseases. It involves replacing a disease-causing gene with a healthy copy, inactivating a defective gene, or adding a new or modified gene. Many gene therapy delivery strategies are available with continuous improvement. Gene therapy for T1D aims to restore insulin production or prevent β cell loss, while it involves improvements in glucose tolerance, insulin resistance, and energy expenditure in T2DM [297]. There are many gene therapy methods involving ex vivo therapy, in vivo therapy, and viral or non-viral vector delivery. Engineering bacterial vectors to prevent infection and deliver therapeutic genes to human cells is under active investigation [281,297]. Non-viral liposomes and nanoparticles deliver DNA or RNA to low-immunogenic cells. Pluripotent stem cells (iPSCs) can be genetically altered to become insulin-producing beta cells via stem cell-based gene therapy. Immune modulation boosts Treg FOXP3 and IL-10, protecting beta cells from autoimmune attacks [297,298].

Current animal studies on diabetic gene therapy provide basic insights and proof of concept for therapeutic applications in humans. In diabetic mice, lentiviruses targeting NeuroD1 and Betacellulin boosted islet neogenesis and insulin production, providing a way to promote endogenous insulin synthesis [298]. In diabetic rats, intraportal INS-lentiviral particle injection maintained hepatic insulin expression and blood glucose levels, suggesting a long-term glycemic management method [281,298]. Intramuscular insulin and glucokinase-expressing AAV (adeno-associated virus) vectors enhanced glucose homeostasis and insulin production in diabetic dogs and mice [281,299]. AAV delivery of INS, PDX1, and GCK improved diabetic mice’s blood glucose management and insulin sensitivity, validating gene therapy’s metabolic regulation [299]. Gene therapy’s revolutionary potential for diabetes treatment is shown in these animal studies, laying the groundwork for human trials and better diabetes outcomes [281,299].

In human gene therapy trials, it has been demonstrated that plasmid VEGF improves neuropathic symptoms and pain in type 1 and type 2 diabetics [300]. Recombinant adeno-associated virus carrying the human insulin gene improved glucose and insulin production over time, suggesting that diabetics may attain sustained glycemic control [300]. In 2023, Kupczynska et al. reported that a bicistronic VEGF165/HGF plasmid enhanced ischemic lesions and angiogenesis in diabetic foot syndrome patients, improving wound management and preventing limb amputation [301]. VM202, an HGF gene therapy, reduced discomfort and ameliorated diabetic peripheral neuropathy [301,302].

Human and animal gene therapy research targeting insulin resistance offers significant prospects for future treatments. Clinical benefits include improved glucose control, insulin production, and considerable pain reduction in diabetes complications. Constant improvement and long-term solutions may reduce the need for pharmacological intervention and improve patient quality of life with this strategy [281,298].

## 12. Personalized Therapies for Insulin Resistance

Treating each patient as an individual with a personalized, tailored approach to treat IR is very appealing. Indeed, very soon we will be able to distinguish biomarkers and apply the appropriate therapy for each patient. These approaches may include stem cell therapy, gene editing using CRISPER (clustered regularly interspaced short palindromic repeats)-based technologies, cell-based, and peptide therapies. This approach has already started with the generation of pancreatic islet transplantation; however, even in patients with long-standing type 1 diabetes, the results are transient. Personalized therapies for insulin resistance require a comprehensive approach that integrates genetic, metabolic, and lifestyle factors. Advances in precision medicine, wearable technology, and AI are enabling more tailored and effective interventions. Collaboration between healthcare providers, dietitians, and patients is essential to achieve optimal outcomes.

## 13. Conclusions

Insulin resistance is a major contributor to metabolic-related diseases like dyslipidemia, T2DM, hypertension, and cancer. It is caused by abnormalities in the insulin signaling cascade, including insulin receptor abnormalities and metabolic disturbances. The global prevalence of diabetes is projected to reach 700 million by 2045, and the rapidly rising incidence of metabolic disease is now regarded as a major global health crisis. Risk factors include genetic, environmental, and lifestyle factors. Childhood obesity, low birth weight, smoking, pollutants, microorganisms, and certain nutritional elements may contribute to IR development. IR rates vary within countries, with urban populations showing the largest rises. The prevalence of IR varies between ethnic groups and depends on socioeconomic status. Lifestyle factors and inherited genetic risk factors could explain prevalence disparities in specific demographic groups, suggesting targeted intervention. In this backdrop, understanding societal and molecular pathways leading to IR is crucial for developing effective treatments, reducing healthcare costs, and improving patient life quality. Increased physical activity is strongly recommended to minimize health consequences of IR. “Exercise snacks” are short, regular workouts that help sedentary people become more active and increase their cardiovascular fitness, metabolic capacity, and muscle function faster and easier than regular exercise. Short and intense exercise snacks mobilize organ systems quickly, boosting skeletal muscle oxygen and glucose consumption, muscle protein synthesis, and other musculoskeletal activities. Balanced normocaloric or hypocaloric diets, depending upon the individual’s requirements, need to be recommended in consultation with the treating physician and the dieticians. Currently used and recently introduced medications target the different aspects of IR and are yielding positive results. However, the associated side effects and the limited availability of some of these therapeutic agents to certain sections of societies are posing a great challenge in this ongoing fight again insulin resistance and its consequences. Emerging therapeutic targets are being investigated to develop novel lead compounds targeting IR and T2DM that are expected to change the landscape of IR treatment.

## Figures and Tables

**Table 1:** Currently used medications against IR.

Role	Drug Class	Examples	Mechanism	Citation
Decrease Hepatic glucose production	Biguanides	Metformin	The precise mechanism of metformin is still elusive and is thought to reduce HGP, a process that is facilitated by the stimulation of mitochondrial activity or the suppression of glucagon signaling via AMPK activation and increased expression of the GLUT4 glucose transporter.	[268]
Increase insulin sensitivity	Thiazolidinediones	Rosiglitazone Pioglitazone	Thiazolidinediones function through their interaction with the PPAR-γ to enhance the sensitivity of adipose muscle and liver to insulin.	[268]
Inhibit renal glucose reabsorption	Sodium-Glucose Cotransporter Inhibitors (SGLT-2i)	Empagliflozin Dapagliflozin	SGLT-2is facilitate insulin-independent glucose reduction by inhibiting glucose reabsorption in the proximal renal tubules, thereby decreasing blood glucose levels. Additionally, These medications are linked to reliable and well documented weight loss and decreases in blood pressure	[268,269]
Increase insulin sensitivity	Glucagon-like Peptide-1 Receptor Agonists (GLP 1 RA)	Semaglutide Dulaglutide Liraglutide Exenatide	GLP 1RAs increase insulin sensitivity in peripheral tissues and also have notable anti-inflammatory and anti-obesity effects, protective benefits for lung health, and favorable impact on gut microbiome composition. However, GLP-1RAs are linked to prevalent gastrointestinal adverse effects, impacting over one-third of patients and other complications.	[268,270]
Increase insulin secretion	Dipeptidyl Peptidase-4 Inhibitors (DPP-4i)	Vildagliptin Alogliptin Linagliptin Gemigliptin Teneligliptin Trelagliptin Saxagliptin	DPP-4is inhibit incretin degradation and facilitates postprandial insulin secretion. Their advantages include the reduction in HbA1c levels, renal microalbuminuria, and inflammation.	[268,271]
Increase insulin secretion	Sulfonylureas	Glimepiride Gliclazide	Sulfonylureas reduce blood glucose levels by enhancing insulin secretion from beta cells through the inhibition of KATP channels. They also inhibit gluconeogenesis and lipid breakdown into fatty acids. They also promote insulin sensitivity.	[268,272]

**Table 2:** Recent drug targets aimed at insulin resistance.

Role	Drug Target	Examples	Mechanism	Citation
Increase insulin secretion	Glucose-dependent insulinotropic polypeptide (GIP)	Tirzepatide	GIP is present in β-cells, adipose tissue, and the brain and increases intracellular cAMP by binding to its receptor. High cAMP levels activate PKA, and exchange protein-activated cAMP2. Depolarizing voltage-gated calcium channels raises intracellular Ca^2+^ and promotes insulin release from β-cells. Recently, Tirzepatide, a novel dual GIP/GLP 1 receptor agonist, not only achieved significantly improved glycemic control but also allowed the majority of participants to attain a mean weight reduction exceeding 10% from baseline, which is a notable outcome in the realm of current pharmacotherapy. Its safety profile is being investigated, and it offers a lot of promise as of now.	[273,274]
↑ Insulin release ↓ HGP	G-Protein coupled receptor (GPCR 119)	GSK1292263, MBX-2982 DS-8500a APD668 BMS-903452	GPR119, a Class-I G protein coupled receptor, is found in muscles, liver, and pancreatic β-cells. Similar to incretin hormones, GPR119 activation may enhance insulin synthesis and secretion when agonists bind to its binding site. GPR119 enhances glucose homeostasis via direct β-cell insulin release and indirect GLP-1 and GIP release in enteroendocrine cells. More than 40 GPR 119 agonists have been reported to show promising effects on glucoses homeostasis by depressing HGP and increasing insulin synthesis in both humans and/or animal models. The efficacy and the safety profile of these agonists is under continuous scrutiny.	[275,276]
↑Incretin hormone Release ↑ Insulin release	Free-fatty acid receptor-1 agonists	TAK-875 TSL1806	G-protein-coupled receptor-40 (FFA1) is a Class-A receptor and is expressed in the mammalian pancreas, gut, taste buds, and CNS. FFA1 affects blood glucose levels by increasing incretin hormones and promoting insulin release from pancreatic β-cells. Synthetic GPR40/FFA1 receptor agonists, such as TAK-875 and TSL1806, have been tried in the last many years, but their side effects, including hepatotoxicity, are a matter of concern, which is being investigated.	[277,278]
Target Fatty acid oxidation	PPAR full agonists	Chiglitazar Sodium	Chiglitazar Sodium is a peroxisome proliferator-activated receptor (PPAR) full agonist simultaneously activates three subtypes of PPAR receptors (α, γ, and δ). It can induce the expression of downstream target genes related to insulin sensitivity, fatty acid oxidation, energy conversion and lipid transport, and inhibit the phosphorylation of PPARγ receptors associated with insulin resistance.	[279]
↑ Insulin release	Melatonin (neuroendocrine hormone)	Melatonin	Melatonin modulates glucose levels via its melatonin receptors MT1 and MT2 in diverse cells. Melatonin supplementation has been reported to ameliorate hyperinsulinemia, insulin resistance, and insulin sensitivity by many investigators and there is enough evidence to use it as an adjuvant therapy.	[280]

## Data Availability

All the data are presented here.

## Abbreviations

T1DM	Type-1 diabetes mellitus
T2DM	Type-2 diabetes mellitus
IR	Insulin resistance
HOMA-IR	Homeostatic model assessment for insulin resistance
IGF	Insulin like growth factor
GRB	Growth factor receptor-bound protein
SHC	Src homology 2 domain-containing adapter protein
CETP	Cholesteryl ester transfer protein
PH	Pleckstrin homology
SH2	Src homology -2
IRS	Insulin receptor substrate
APS	Adapter protein with a PH and SH2 domain
Ras	Rat sarcoma virus oncogene
MAPK	Mitogen activated protein kinase
IRR	Insulin related receptor
IGF-1R	IGF-1 receptor
mMRA	messenger RNA
IR-A	Insulin receptor-A
KO	knockout
ERK	Extracellular signal-regulated kinase
DOK4	Docking protein4
GTP	Guanosine triphosphate
GAB	Grb2-associated binder
Cbl gene	Casitas B-lineage Lymphoma gene
CAP	catabolite activator protein
DOK	Docking protein
PI3K	Phosphatidylinositol 3-kinase
AKT	serine/threonine-protein kinase also known as protein kinase B
Pik3r1	Phosphoinositide-3-Kinase Regulatory Subunit 1
Src	Steroid receptor coactivator
Csk	C-Terminal Src Kinase
DOCK	Dedicator of cytokinesis protein
Crk	Proto-oncogene c-Crk protein
PKB	Protein kinase B
mTORC	Mammalian target of rapamycin complex 1
DNAPK	DNA-dependent protein kinase
FOXO1	Forkhead box protein O1
TBC1D4	TBC1D4 (TBC1 Domain Family Member 4)
PGC	Peroxisome proliferator-activated receptor-gamma coactivator
PDE3B	PDE3B phosphodiesterase 3B
c-AMP	Cyclic adenosine monophosphate
Cip 1	Cdk-interacting protein-1
WAF 1	wildtype p53-activated fragment 1
p27Kip1	Cyclin-dependent kinase inhibitor 1B
IKK	IκB kinase
PKC	Protein Kinase C
nPKCs	Novel protein kinases
aPKCs	atypical protein kinases
SREBP1	Sterol regulatory element-binding protein 1
SOS	Son of Sevenless (a set of genes)
MEK	Mitogen-activated protein kinase kinas
PTP1B	Protein tyrosine phosphatase 1B
LAR	leukocyte common antigen-related protein
PP2A	Protein Phosphatase 2A
PP2B	Protein Phosphatase 2B
S6K	S6 kinase p70
PHLPP-1	PH domain leucine-rich repeat protein phosphatase 1
PTEN	Phosphatase and tensin homolog
SHIP	SH2 domain-containing inositol 5-phosphatases
SOCS	Suppressor of Cytokine Signaling
IP7	Inositol pyrophosphate
IP6K1	Inositol hexakisphosphate kinase 1
Trb3	Tribbles homolog 3
JNK	c-Jun N-terminal kinase
Ser-307	Serine residue at position 307
DAG	Diacylglycerol
PKA	Protein kinase A
PPARγ	Peroxisome proliferator-activated receptor-γ
GLUT	Glucose transporter
GAP	GTPase-activating protein
RAC-1	Ras-related C3 botulinum toxin substrate 1
GYS	Glycogen synthase
GSK	Glycogen synthase kinase
IRTK	Insulin-Induced Receptor Tyrosine Kinase
HGP	Hepatic glucose production
G6PC1	Glucose-6-phosphatase catalytic subunit 1
PEPCK	Phosphoenolpyruvate carboxylase
SREBP-1	Sterol regulatory element-binding protein
ACC1	Acetyl-CoA carboxylase 1
GPAT1	Glycerol-3-phosphate acyltransferase
NAFLD	Non-alcoholic fatty liver disease
ROS	Reactive oxygen species
ER	Endoplasmic reticulum
NFκB	Nuclear factor kappa B
TLR4	Toll-like receptor 4
CerS	Ceramide synthase
FFA	Free fatty acids
MCP-1	Monocyte chemoattractant protein-1
TNF- α	Tumor necrosis factor alpha
IL	Interleukin
CLS	Crown like structure
JAK-STAT	Janus kinase signal transducer and activator of transcription
NOX	NADPH oxidase
GPX	Glutathione peroxidase
Mfn1	Mitofusin1
Drp1	Dynamin-related protein 1
VLDL	Very low-density lipoprotein
DAMP	Damage-associated molecular patterns
ULK1	Unc-51 like autophagy activating kinase 1
SERCA	Sarcoendoplasmic reticulum calcium transport ATPase
TFEB	Transcription factor EB
PC	Phosphatidylcholine
PERK	Protein kinase R like protein kinase
ATF	Activating transcription factor
IRE-1	Inositol-requiring enzyme type 1
XBP1	X-box binding protein 1
F25BS	Los Angeles insulin
F25BL	Chicago insulin
PTPN1	Protein tyrosine phosphatase N1
LDLR	Low density lipoprotein receptor
IGF1R	Insulin-like growth factor receptor-1
AgRP	Agouti-related protein
POMC	Pro-opiomelanocortin
Ins1	Insulin 1 gene
VEC	Vascular endothelial cell
VCAM	Vascular cell adhesion molecule
eNOS	endothelial nitric-oxide synthase
iNOS	Inducible nitric-oxide synthase
CVD	Cardiovascular disease
SLI	silent lacunar infarction
Ty-G	Triglyceride-glucose index
END	Early neurological degeneration
PCOS	Polycystic ovarian syndrome
INSR	Insulin receptor
NALP3	Nucleotide-binding domain, leucine-rich repeat/pyrin domain-containing-3
